# Toward a more comprehensive modeling of sequential lineups

**DOI:** 10.1186/s41235-022-00397-3

**Published:** 2022-07-22

**Authors:** David Kellen, Ryan M. McAdoo

**Affiliations:** Department of Psychology, Syracuse University, Syracuse, NY USA

## Abstract

Sequential lineups are one of the most commonly used procedures in police departments across the USA. Although this procedure has been the target of much experimental research, there has been comparatively little work formally modeling it, especially the sequential nature of the judgments that it elicits. There are also important gaps in our understanding of how informative different types of judgments can be (binary responses vs. confidence ratings), and the severity of the inferential risks incurred when relying on different aggregate data structures. Couched in a signal detection theory (SDT) framework, the present work directly addresses these issues through a reanalysis of previously published data alongside model simulations. Model comparison results show that SDT modeling can provide elegant characterizations of extant data, despite some discrepancies across studies, which we attempt to address. Additional analyses compare the merits of sequential lineups (with and without a stopping rule) relative to showups and delineate the conditions in which distinct modeling approaches can be informative. Finally, we identify critical issues with the removal of the stopping rule from sequential lineups as an approach to capture within-subject differences and sidestep the risk of aggregation biases.

Since its formation in 1992, the Innocence Project (2021) has exonerated 232 people wrongfully convicted of a crime. Out of these successful exonerations, 63% involved mistaken eyewitness identifications from a lineup procedure. Given these numbers, it is important to understand the factors that influence mistaken identifications in lineups, and evaluate the benefits and shortcomings that are associated with different procedures. Research into the causes of mistaken identifications has a long history in social and cognitive psychology (for a review, see Gronlund & Benjamin, 2018). However, it is only within the past decade or so that researchers began to make use of some of the formal modeling approaches available in their toolboxes (e.g., Goodsell et al., 2010; Palmer & Brewer, 2012; Wetmore et al., 2017; Wixted et al., 2018).

The present work extends these endeavors to the case of *sequential lineups*, a procedure that has been the subject of a large number of empirical investigations (for a review of 72 such tests, see Steblay et al., 2011b), but comparatively few theoretical, model-driven ones (e.g., Carlson et al., 2016; Dunn et al., 2010; Horry et al. 2012; Kaesler et al., 2020; Palmer & Brewer, 2012; Wetmore et al., 2017; Wilson et al., 2019). The main motivation behind the present work is the realization that previous model-based analyses of the sequential lineups have failed to preserve the sequential nature of the judgments produced at level of the data (e.g., Kaesler et al., 2020; Wilson et al., 2019) or the models themselves (e.g., Carlson et al., 2016; Horry et al., 2012). As it will become clear below, these issues are not inconsequential, as they can severely distort results.

The goal of the present work is to address these issues, bridge a number of existing knowledge gaps, and raise new concerns. This general goal will be achieved by applying a large family of models based on *Signal Detection Theory* (SDT) to the large-scale sequential lineup data reported by Wilson et al. (2019) and Dunn et al. (2022). These models will allow us to test a number of hypotheses regarding people’s discriminability and response bias throughout the sequential lineup procedure. They will also provide us with the necessary backdrop for addressing a number of issues, from the general ability to characterize sequential lineup data with SDT, to the inferential risks associated with the use of aggregate data.

The remainder of this manuscript is organized as follows: First, we will provide a brief introduction to sequential lineups and discuss how sequential lineup data have been modeled using SDT up to this point. This introduction will be followed by a reanalysis of Wilson et al.’s and Dunn et al.’s data, along with discussions on the different issues that arise in the context of each study. Altogether, our model analyses showcase the benefits of modeling sequential lineups with SDT—but also show how challenging the characterization of lineup data can be.

## Sequential lineups

Experiments simulating lineup identifications follow a fairly standard format. Participants are shown a mock crime (usually in the form of a video) and are then presented a photograph array of (typically) six faces. The arrays either contain the face of the perpetrator (guilty suspect) of the mock crime or the perpetrator is replaced by an innocent lure. These arrays are referred to as *target-present* and *target-absent* lineups, respectively. When presented with either a target-present or a target-absent lineup, participants can produce the following response outcomes:^1^

- *Target-Present Lineups*:
  - *Outcome TP1*: Correct identification of the guilty suspect (*Hit*),
  - *Outcome TP2*: Incorrect identification of a lure (*False Alarm*),
  - *Outcome TP3*: Incorrect rejection of the lineup (*Miss*),
- *Target-Absent Lineups*:
  - *Outcome TA1*: Incorrect identification of a *known* lure (*False Alarm*),
  - *Outcome TA2*: Incorrect identification of a lure *standing for the designated innocent suspect* (*False Alarm*),
  - *Outcome TA3*: Correct rejection of the lineup (*Correct Rejection*).

By comparing these outcomes across different procedures and/or conditions, researchers are able to draw conclusions as to how various factors (such as lineup presentation, instructions) may affect identification performance.

Sequential lineups are among the most commonly used eyewitness identification procedures. In a sequential lineup, faces are presented one at a time to an eyewitness, with each face being subjected to a binary “yes” or “no” judgment at the time of their presentation. If no face is recognized by the time the last face is shown (i.e., the witness responded “no” to all faces), the lineup is said to be rejected. In the USA, for our purposes, a sequential lineup is typically terminated once a “yes” decision is made on a single face. This *stopping rule*, along with the impossibility of revising previous “no” judgments, can be expected to affect people’s reluctance to make a positive identification at any given point. But this reluctance might change across the sequence, perhaps due to the prospect that no identification might be made by the time the sequence ends (see Baumann et al., 2020; Lee & Courey, 2021). This possibility is corroborated by numerous eyewitness identification studies reporting sequence position effects (Goodsell et al., 2010; Gronlund et al., 2009; Meisters et al., 2018; Neuschatz et al., 2016).

The distinctive characteristics of sequential lineups (e.g., single-face presentation, stopping rule) have motivated researchers to compare them with other procedures, such as *simultaneous lineups* (in which all faces are shown at once) or the *showup* procedure (where only one face is presented in the entire procedure). Lindsay and Wells (1985) were among the first to directly compare the performance of simultaneous and sequential lineups, concluding that sequential lineups provided better protection against lure (innocent suspect) identifications in target-absent lineups than simultaneous lineups did, with little effect on guilty suspect identifications in target-present lineups. Meta-analyses by Steblay et al. (2001) and Steblay et al. (2011b) reported that sequential lineups consistently produced similar correct identifications and lower false identifications when compared to simultaneous lineups. More recently, however, the superiority of sequential lineups over simultaneous lineups has been called into question, with researchers claiming that sequential lineups do not induce more accurate identifications, just less identifications overall (for a review, see Gronlund et al., 2015). A parallel discussion has focused on the comparison between different kinds of sequential lineup procedures and the impact of enforcing multiple viewing laps before a decision is made (e.g., Steblay et al., 2011a; Horry et al., 2015; Seale-Carlisle et al., 2019). Regardless of the claims being made in these debates, one thing is clear: The comparison between different eyewitness identification procedures requires a formal characterization of the mnemonic and decision-making processes taking place in each of them. But if these formal characterizations are to inform practices in the US justice system and beyond, then it is necessary to evaluate their theoretical and empirical merits in a comprehensive manner.

### Models of sequential lineup identifications

**Fig. 1:**
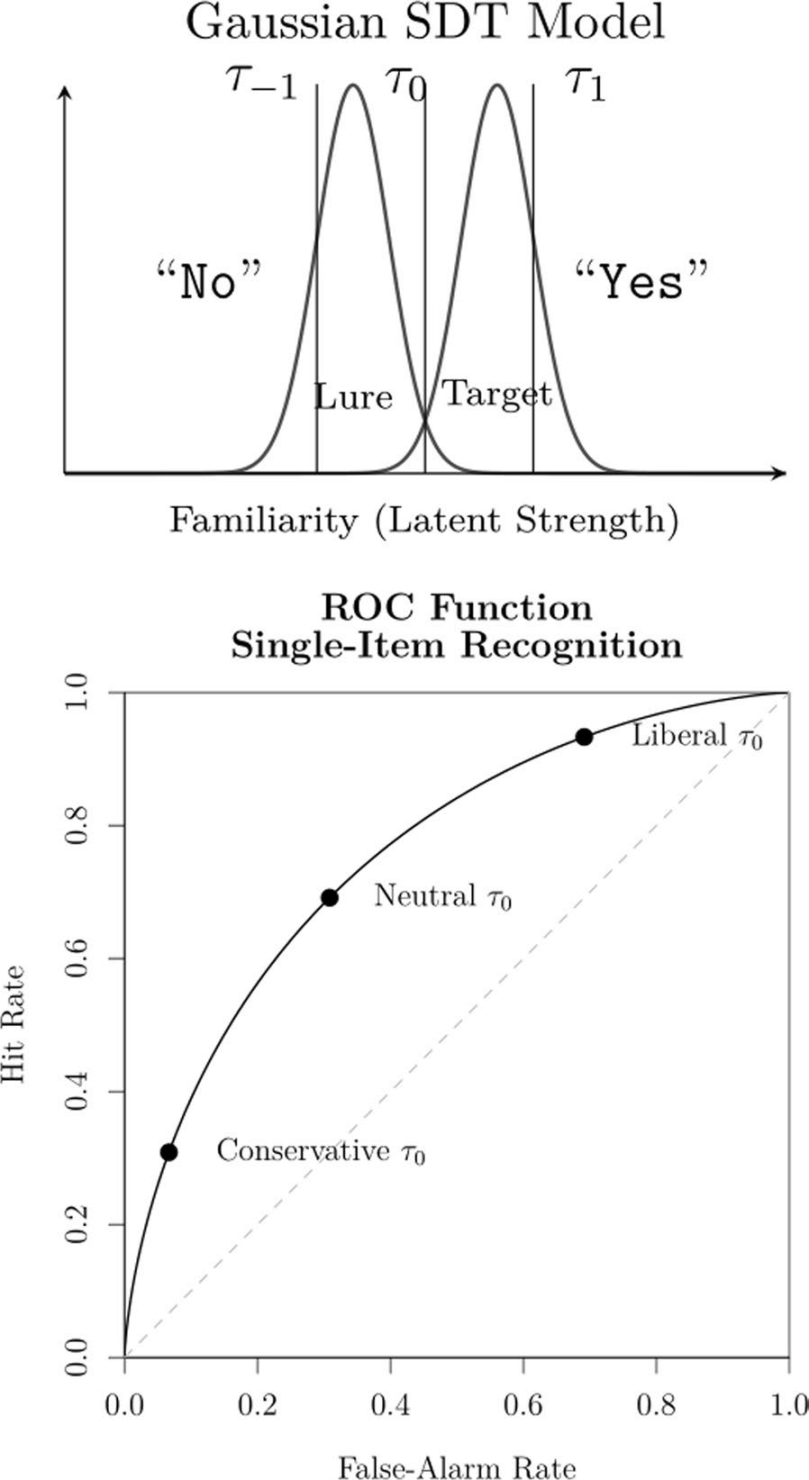
Top panel: Illustration of the Gaussian SDT model. The vertical lines represent the response criterion $\tau _0$ and the confidence criteria $\tau _{-1}$ and $\tau _1$. Bottom panel: Example of a ROC function for the case of single-item recognition

Recent modeling efforts in the field of eyewitness identification have been couched on signal detection theory (SDT; Green & Swets, 1966; Kellen & Klauer, 2018; Macmillan & Creelman, 2005). According to SDT, the classification of different classes of stimuli (e.g., faces) can be characterized in terms of a comparison between the *latent strength* values of the stimuli and a *response criterion*. In the context of recognition memory—and eyewitness identification—when a stimulus is presented to a decision-maker, she compares the latent memory strength (also referred to as familiarity) of that stimulus with a previously established response criterion $\tau _0$. If the memory strength is greater than the criterion, an affirmative response is made, such as “old” or “yes.” If it is below the criterion, then a negative response such as “new” or “no” is produced. The value taken by the response criterion indicates how conservative/liberal the decision-maker is, with larger values indicating greater conservatism (i.e., reduced willingness to respond “yes”). Different classes of stimuli (targets versus lures) are described in terms of different latent strength distributions. The top panel of Fig. 1 provides an illustration, in which the latent strength distributions are assumed to be Gaussian. The ability to discriminate between two classes of stimuli is represented by their relative distance (i.e., their degree of overlap). The greater the distance (i.e., the smaller the overlap), the more discriminable the two stimulus classes are. Beyond binary (i.e., yes/no; old/new) responses, SDT can characterize responses on a confidence rating scale (e.g., ranging from “very sure no” to “very sure yes”) by introducing a set of ordered confidence criteria, such as the $\tau _{-1} \le \tau _0 \le \tau _1$ criteria illustrated in Fig. 1. Varying the binary criterion $\tau _0$ (or taking the confidence criteria as its proxies) leads to joint changes in the hit and false alarm rates, a functional relationship commonly known as a *Receiver Operating Characteristic* (ROC) function. The bottom panel of Fig. 1 provides an example of a ROC function.

One of the key benefits of modeling data with SDT is the fact that it provides us with a principled way to test a number of substantive hypotheses. More specifically, it allows us to test whether differences in observed responses can be attributed to (1) differences in discriminability, (2) shifts in the response criterion, (3) or both. This ability, which cannot be achieved by means of naïve summary statistics (see Rotello et al., 2015), has been put to use in eyewitness identification research (Dunn et al., 2022; Kaesler et al., 2020; Wilson et al., 2019; Wixted et al., 2017). Many of these SDT model implementations have focused on testing a prominent hypothesis in eyewitness research known as the *diagnostic feature detection* (DFD) hypothesis (Wixted & Mickes, 2014).

The DFD hypothesis argues that participants can use the features of the different faces in the lineup to learn how to better discriminate between targets and lures. In SDT terms, this learning process is expected to reduce the overlap between the latent strength distributions for these stimulus classes (Wixted & Mickes, 2014). In the case of *simultaneous lineups*, witnesses view and directly compare multiple faces before making a judgment. This situation allows them to identify and ignore the features that are similar among all the faces (e.g., brown hair, if all lineup members have it) and focus in on unique, or diagnostic, features that can provide the most aid in making a correct decision (e.g., the specific shape of the perpetrator’s eyes). This opportunity to learn how to better discriminate is *partially* compromised in the case of sequential lineups, given that witnesses only get to see one face at a time. At most, the witness can learn about the diagnosticity of features as the lineup sequence unfolds. However, there is the risk that the target is missed when presented earlier on, as well as the risk that the witness will incorrectly identify someone before having the opportunity to encounter the target. There is also the issue that memory discriminability tends to diminish along a sequence of judgments (e.g., Criss et al., 2011; Osth et al., 2018), a phenomenon that, if present in the context of sequential lineups, might counteract the improvements predicted by the DFD hypothesis.

Because of the different learning opportunities that alternative procedures provide, the DFD hypothesis expects performance in simultaneous lineups to be better than in sequential lineups, as well as showups. This prediction has become the subject of much empirical discussion recently (e.g., Carlson et al., 2019; Carlson et al., 2021; Colloff & Wixted, 2020; Kaesler et al., 2020; Smith et al., 2017; Wetmore et al., 2017; Wixted & Mickes, 2015). In the case of sequential lineups, the DFD hypothesis *expects discriminability to increase across sequence positions*. This prediction was recently tested by Wilson et al. (2019), who compared discriminability in the first sequence position with all the other positions. Their results were consistent with the predictions of the DFD hypothesis. A more recent experiment reported by Dunn et al. (2022) also produced consistent results.

### Beyond differences in discriminability

Although the kind of learning postulated by the DFD hypothesis is expected to be somewhat limited in the case of sequential lineups, other kinds of learning are nevertheless possible. For instance, witnesses might learn about their ability to discriminate between targets and lures *and use this knowledge to adjust the response criteria accordingly* (e.g., Brown & Steyvers, 2005; Brown et al., 2007; Osth & Dennis, 2015; Stretch & Wixted, 1998). In a seminal paper, Treisman and Williams (1984) showed how a number of sequential dependencies found in people’s judgments can be attributed to trial-by-trial shifts in response criteria (see also Kac, 1962; Vickers & Lee, 1998). These shifts are governed by a learning process that is sensitive to the latent strengths that were recently encountered, as well as long-term goals, such as maintaining a response criterion that imposes a certain amount of conservatism (e.g., Kantner & Lindsay, 2012, 2014), or having a criterion that tracks a changing environment (e.g., Baumann et al., 2020; Lee & Courey, 2021).

More recently, Turner et al. (2011) showed how individuals can gradually learn about different latent strength distributions and use this continuously updated knowledge to maintain a certain level of response bias. Their account is particularly relevant in the case of sequential lineup procedures, given the small number of judgments that witnesses can make, and the fact that a priori, the witness is uncertain of how difficult it will be to distinguish the target from the lures (e.g., how similar they are to the target). According to Turner et al., participants begin by relying on their prior beliefs about how familiar targets and lures are expected to be. These prior beliefs can be easily constructed by the participant/eyewitness using a handful of sampled exemplars (for an overview, see Chater et al., 2020).

**Fig. 2:**
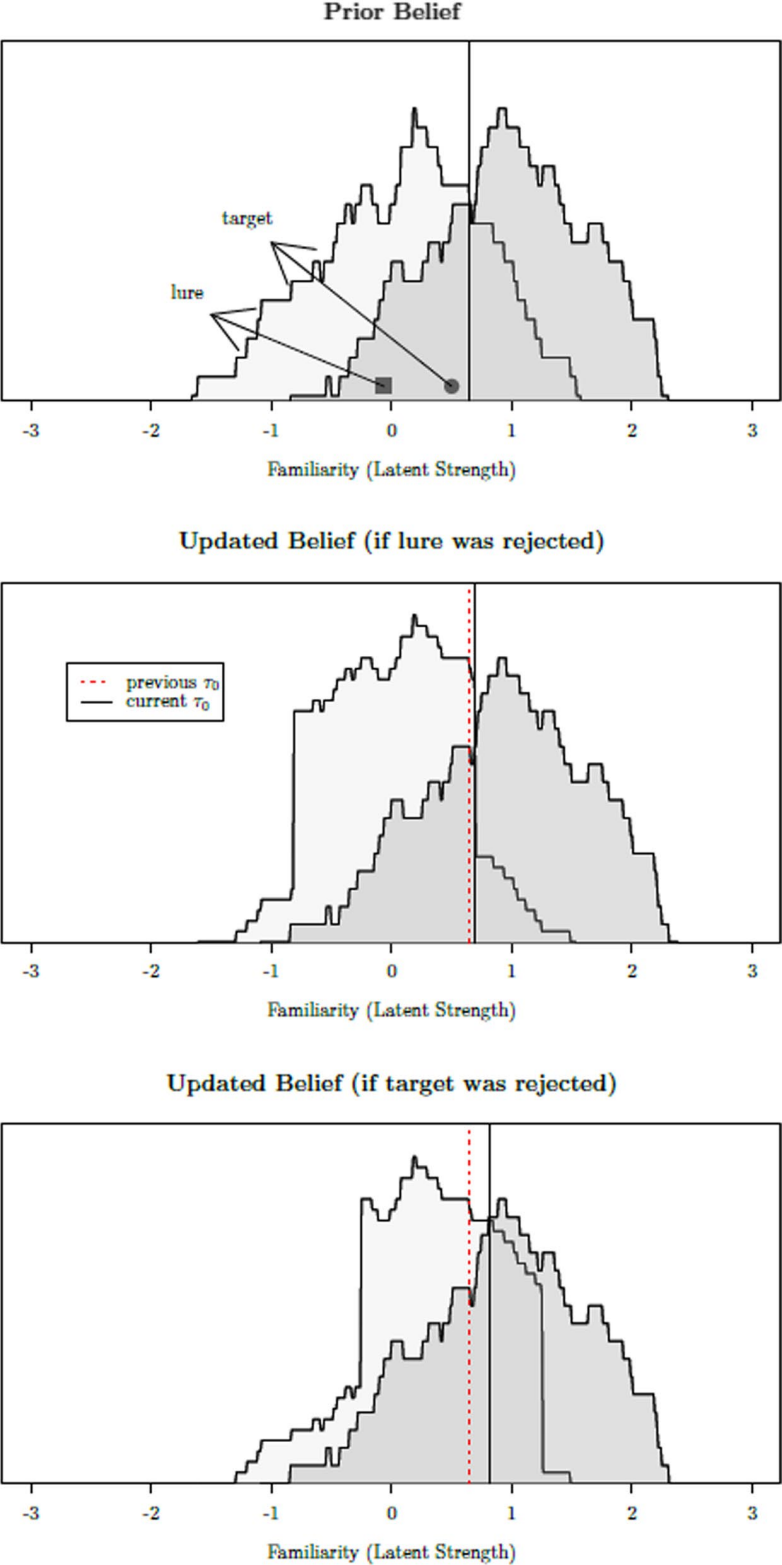
Top panel: The *believed* distribution of latent strength values for targets (darker gray) and lures (lighter gray) up to the point in which the target (solid circle) or a lure (solid square) are encountered. Note that both latent strengths are below the response criterion (solid black line), which is set at the point of equal likelihood (i.e., where both distributions intersect). Middle panel: The updated *believed* distributions of latent strength values if the lure was encountered and rejected. Bottom panel: The updated *believed* distributions of latent strength values if the target was encountered and rejected instead. In both the middle and bottom panels, the prior response criterion (dotted red line) is given for reference

To better understand the learning processes proposed by Turner et al. (2011), let us consider the toy example illustrated in Fig. 2. The prior belief representations for targets and lures, illustrated in the top panel of Fig. 2, are updated every time an item is judged. More specifically, the latent strength of the just-judged item is used to update the target or the lure distribution, depending on how the item was judged (i.e., old or new). Based on previous modeling work, we expect the expected latent strength of targets to be substantially greater than that of lures (e.g., Wixted et al., 2018). One immediate consequence of this fact is that, among the rejected items, targets will generally have greater latent strengths than lures (for a formal proof, see Kellen & Klauer, 2014). If we take into consideration the aforementioned studies showing how response criteria are a function of people’s beliefs regarding targets and lures, then it follows that the *response criterion is expected to increase after the rejection of the target*. This shift is illustrated in the panels of Fig. 2:^2^ In the top panel of Fig. 2, the response criterion is set to 0.65, the value at which the two distributions intersect (likelihood ratio is 1). Now let us consider two scenarios: One in which a lure is presented (solid square) and another where the target is presented (solid circle). Note that both items are below the response criterion and therefore will be rejected. The middle panel illustrates an updated lure distribution (target distribution remains the same) for the scenario in which the lure was rejected. As you can see, the change in the criterion is now set at 0.69, a barely noticeable difference. The bottom panel, which illustrates the updated lure distribution when the target was rejected instead, shows a much more considerable shift rightwards: The criterion is now 0.81, the new value at which both distributions intersect.^3^ Effects of this kind are expected to be small in typical recognition memory experiments, given that participants engage in many dozens if not hundreds of test trials (for a recent empirical investigation, see Malmberg & Annis, 2012). The situation is quite different in most sequential lineups, with witnesses encountering up to six faces at most.

The possibility that different kinds of learning are taking place is something that should not be overlooked or downplayed. When specifying alternative models, researchers should try to develop a family of candidate models that covers all the different possibilities: *are all, some, or none of these kinds of learning taking place?* Failing to consider these different possibilities can lead to distorted results. This issue was recently raised by van den Berg et al. (2014) in the domain of visual working memory. They showed that the evidence for certain hypotheses (e.g., *is the number of remembered items fixed?*) can depend on how exactly the models being used happen to address other substantive issues (e.g., *is working memory precision quantized?*). In the case of sequential lineups, it is possible that the evidence for the DFD hypothesis that was originally reported by Wilson et al. (2019) is dependent on the constraints that they imposed at the level of response criteria. In order to address this issue, one needs to consider a large family of SDT models that includes a factorial combination of hypotheses concerning discriminability and response criteria.

### Conditioning in sequential lineup data

**Fig. 3:**
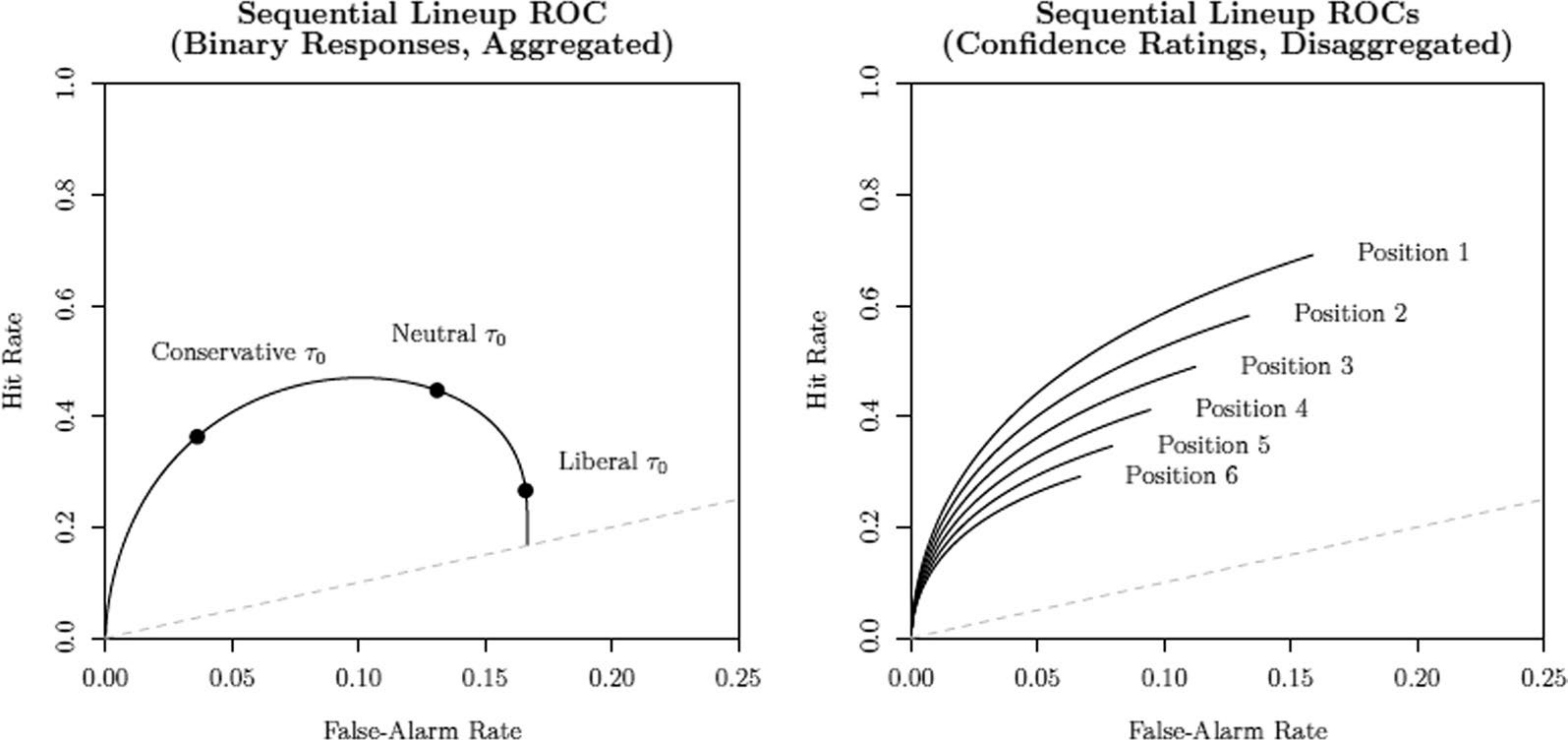
Example of ROCs obtained in a sequential lineup procedure. Left panel: The predicted ROC function when varying response criterion $\tau _0$ and aggregating across sequence positions. Right panel: Confidence rating ROCs obtained for each sequence position

The unique structure of sequential lineup data introduces interesting challenges in its comparison with other procedures and in the testing of hypotheses like the DFD. Typically, one could determine whether or not there are differences in discriminability between procedures by comparing the ROC functions constructed from the eyewitnesses’ confidence rating judgments. A ROC function plots the relationship between false alarms (usually calculated by dividing the filler identification rate by the number of faces in the lineup) and hits in target-absent and target-present lineups, respectively. But as shown by Rotello and Chen (2016), the sequential lineup has unique features that preclude such an approach. In the case of single-stimulus recognition like a showup or simultaneous lineup, where only one final decision is required, changes in response criterion $\tau _0$ (e.g., becoming more conservative or liberal) affect the expected pair of hit and false alarm rates. All possible pairs of values define a *single monotonic* ROC function (see the bottom panel of Fig. 1). This simple relationship no longer holds in the case of a sequential lineup procedure: A more liberal criterion will likely lead to a “yes” response at one of the earlier sequence positions, often before the target is encountered. In contrast, a more conservative criterion is likely to lead to no face or item being accepted at all. Unlike the ROC functions obtained in the case of single-item recognition, changes in response criteria in a sequential procedure yield a *non-monotonic* ROC function, as shown in the left panel of Fig. 3 (compare it with the bottom panel of Fig. 1). Moreover, as shown by Wilson et al. (2019), the confidence ratings associated with “yes” responses at different target sequence positions yield a *family of ROC functions* (see the right panel of Fig. 3).

A key insight stemming from Rotello and Chen’s (2016) work is that one should not overlook the peculiarities of sequential lineup data. This point was furthered by Wilson et al. (2019) who showed that the confidence ratings obtained with such procedures yield a family of ROC functions when disaggregated by target position. These insights tell us that it is unwise to aggregate confidence ratings across sequence positions, especially when comparing ROCs obtained with different procedures. Doing so not only leads to a distorted representation of discriminability (e.g., an underestimation of the ROCs associated with earlier sequence positions), but also overlooks the existing interplay between the different ROCs. After all, the overall response frequencies obtained across the different sequence positions are also a function of discriminability and response criteria as established by the SDT model.

Unfortunately, the important lessons found in Rotello and Chen (2016) are not entirely reflected in some of the more recent attempts to model sequential lineups. Even though researchers have begun to establish models that explicitly capture the peculiarities of sequential lineups, the kinds of data structures that they often rely on still obfuscate them (e.g., Kaesler et al., 2020; Wilson et al., 2019). To see this more clearly, let us consider a sequential lineup procedure in which sequences of *I* faces are presented. Assuming that lure faces are exchangeable, there are $I+1$ possible sequences: namely the *I* *target-present lineup* sequences in which the target takes position *i*, plus one *target-absent* lineup sequence. If we only consider binary responses, the data from this procedure are comprised of $I+1$ “category systems” with a total of $(I+1)\times I$ degrees of freedom (i.e., non-redundant categories). Consider the data from Experiments 1 and 2 from Wilson et al. (2019), reported together in Table 1: Their lineup procedure, with sequences of $I=6$ faces, yields a $7\times 7$ data matrix (each of the seven columns corresponding to a category system) with $7\times 6 = 42$ degrees of freedom. The frequencies reported in each column of Table 1 correspond to responses conditioned on *lineup type and sequence*. If we decide to unpack “yes” responses in terms of *K* *levels of confidence*, then each category system is comprised of $(I+1)\times (I \times K + 1)$ categories, yielding a total of $(I+1)\times I \times K$ degrees of freedom.

**Table 1:** Choice frequencies from Experiments 1 and 2 of Wilson et al. (2019) and Dunn et al. (2022)

Binary “yes”/“no” Judgments							
Wilson et al. (2019)							
	Target position						
Response	1	2	3	4	5	6	Absent
“yes” to Position 1	**579**	137	127	123	132	125	766
“yes” to Position 2	*2*	**450**	98	82	91	93	539
“yes” to Position 3	*9*	*7*	**391**	108	109	89	554
“yes” to Position 4	*14*	*15*	*9*	**331**	67	62	488
“yes” to Position 5	*21*	*7*	*5*	*2*	**229**	53	305
“yes” to Position 6	*9*	*6*	*9*	*8*	*5*	**214**	232
Reject all	75	74	52	45	63	65	**1267**
Total	709	696	691	699	696	701	4151

Dunn et al. (2022)							
	Target position						
Response	1	2	3	4	5	6	Absent
“yes” to Position 1	**701**	119	104	122	90	106	185
“yes” to Position 2	*8*	**626**	68	75	68	62	94
“yes” to Position 3	*7*	*10*	**631**	72	47	91	99
“yes” to Position 4	*11*	*10*	*12*	**532**	63	63	86
“yes” to Position 5	*17*	*12*	*6*	*6*	**485**	53	87
“yes” to Position 6	*15*	*12*	*11*	*9*	*10*	**421**	79
Reject all	224	185	175	154	148	143	**790**
Total	983	974	1007	970	911	938	1420

The frequencies in bold font correspond to the correct responses. The frequencies in italic correspond to acceptances of a lure after the rejection of the target. The underlined frequencies (when expanded into confidence levels) are those used in the plotting of receiver operating characteristic (ROC) functions (see the right panel of Fig. 3). Please note that the frequencies from Wilson et al. (2019) exclude 153 participants with missing data

Wilson et al. (2019) relied on a different conditioning when structuring their data. Specifically, they conditioned on *lineup type and sequence* *position*. This means that for each position *i*, we have a category system for each type of sequential lineup. In the case of target-present lineups, we have three categories: 1) “yes” judgments to targets, 2) “yes” judgments to lures, and 3) “no” judgments. In the case of target-absent lineups, we have “yes” and “no” responses to lures. Under this conditioning, binary “yes”/“no” judgments yield $I\times 2$ category systems with a total of $I \times (2 + 1)$ degrees of freedom. If we unpack “yes” judgments into *K* levels of confidence, we end up with a total of $I \times (2 + 1) \times K$ degrees of freedom. In the case of Wilson et al. (2019), partitioning “yes” responses into five levels of confidence resulted in a total of $6 \times (2+ 1) \times 5 = 90$ degrees of freedom (see their Table 4). Note how this number is less than half of the 210 degrees of freedom that would be obtained if one conditioned on *lineup type * and *sequence* instead. This reduction in degrees of freedom indicates that some information is being lost. For instance, note that “yes” judgments to lures, conditional on position *i* in a target-present lineup, aggregate responses across all target-present lineups in which the target was present in positions $j\ne i$. This aggregation compromises our ability to observe any differences between alternative sequences. Moreover, it forces the data to include the same single responses multiple times across the different category systems, which violates their (functional) independence.^4^

### Confidence ratings

Confidence ratings play an important role in SDT modeling (Green & Swets, 1966; Kellen & Klauer, 2018; Macmillan & Creelman, 2005), especially in its application to recognition memory (for recent reviews, see Rotello 2018, Wixted, 2020). They also play an increasingly vaunted role in the eyewitness literature (Wixted & Wells, 2017). The ROC functions that can be produced from confidence ratings have allowed recognition memory researchers to obtain more “refined” SDT characterizations (i.e., all model parameters are estimated) that would not be possible on the basis of binary judgments unless certain experimental manipulations and selective influence assumptions were introduced (see Bröder & Schütz, 2009; Dube & Rotello, 2012). Given that the application of SDT modeling in the context of eyewitness identification is largely informed by its impressive track record, especially in the domain of recognition memory, it is not surprising to see a similar reliance on confidence ratings (e.g., Gronlund, et al., 2012; Gronlund et al., 2014; Wetmore et al., 2017; Wixted & Mickes, 2012; 2018). However, it is important to keep in mind that the challenges faced in these two domains are not quite the same—the data obtained from binary judgments in a sequential lineup are much richer. To see this, simply note that the (disaggregated) data coming from binary judgments in a sequential lineup, such as the one conducted by Wilson et al. (2019; see Table 1), yield *twenty-one times more degrees of freedom* (42 vs. 2).

These differences suggest that confidence ratings might be unnecessary when it comes to modeling sequential lineups with SDT.^5^ The possibility of a “streamlined” SDT application based on binary judgments is interesting in the sense that it could potentially alleviate some of the misfits that have been reported so far (e.g., Kaesler et al., 2020, Wilson et al., 2019). After all, the sequential lineup data being modeled arise from the aggregation of responses from multiple eyewitnesses, each with their own discriminability and response criteria. It is well known that the aggregation of said responses can introduce distortions that might not be well handled by the *parametric* SDT model being used (e.g., Kellen & Singmann, 2016; Morey et al., 2008; Rabe et al., 2021; Trippas et al., 2018). The fact that individuals can differ in the way that they engage with rating scales is likely to exacerbate this issue (see Hamilton, 1968; Henninger & Meiser, 2020; Tourangeau et al., 2000). We will therefore extend our modeling to confidence judgments following a successful implementation to binary judgments.

## General modeling approach

In the analyses below, we will develop and test a large body of SDT models, each reflecting the different ways in which memory judgments can be affected by sequence position: One general hypothesis is that memory discriminability is affected by sequence position, with the DFD hypothesis postulating that it should increase (Wilson et al., 2019; Wixted et al., 2017). Another hypothesis is that the positioning of the response criteria is not stable, being affected by the specific history of faces encountered by each eyewitness throughout the sequential lineup. Based on the modeling insights by Treisman and Williams (1984) and more recently Turner et al. (2011), there is good reason to assume that response criteria, especially the criterion responsible for binary judgments, will increase after the rejection of the target (see also Dunn et al., 2022).

The different SDT models will be fit to the large-scale datasets reported by Wilson et al. (2019) and Dunn et al. (2022). Each of these studies will be considered in turn. The reason behind this “sequential” organization is that, beyond directly comparing different candidate models, we also report additional analyses addressing issues that are especially relevant in the context of each study.

In the case of Wilson et al. (2019), we are dealing with the first large-scale study that permits a full-fledged implementation of SDT models tailored to the sequential lineup procedure. This seminal status calls for an expanded set of analyses that address a number of foundational modeling questions:

- **Q1:** Can we obtain adequate SDT characterizations from binary/confidence judgments?
- **Q2:** What are the limits of binary judgments in terms of estimation and hypothesis testing?
- **Q3:** What is the ability to detect differences in discriminability as postulated by the DFD hypothesis? Is this ability a function of the flexibility given to response criteria?
- **Q4:** What are the merits of sequential lineups, relative to other procedures such as showups? And what is the impact of imposing a stopping rule?

The follow-up study by Dunn et al. (2022), aside from reporting large study that attempts to replicate the results of Wilson et al. (2019) while imposing an actual stopping rule, raised a number of important issues regarding the use of aggregate data and its inferential risks. Our analyses directly address these issues and provide answers to the following questions:

- **Q5:** Can aggregation biases produce spurious support for learning (in terms of discriminability and/or response criterion) throughout the sequential lineup?
- **Q6:** Can aggregation biases be sidestepped by removing the stopping rule from the sequential lineup procedure?

## Revisiting Wilson et al. (2019)

In the two experiments reported by Wilson et al. (2019), participants engaged in a sequential lineup procedure. For each face presented, participants were asked whether it corresponded to the guilty suspect as well as their confidence in that judgment (using a scale ranging from − 100 to 100). Importantly, Wilson et al.’s procedure did *not* include a stopping rule, which meant that participants always evaluated every single face in the lineup. In order to address this deviation from standard practices, we excluded any participants’ responses beyond their first “yes” judgment; i.e., we emulated a stopping rule. Because both experiments used the same stimuli and implemented the exact same lineup procedure, we were able to collapse participants’ responses into a single dataset, which is reported in Table 1.

### Analysis of binary judgments

The SDT modeling of sequential lineup judgments can be cast as an extension of a yes–no task (for a previous treatment, see Kaesler et al., 2020). Let $S=s$ denote a given sequence of *I* faces that can include the target (guilty suspect) or not. For each position *i* in the sequence, with $1 \le i \le I$, the witness is requested to judge whether or not they recognize the face being presented to them. According to SDT, each presented face is associated with latent strength or familiarity value that was sampled independently from a target distribution or a lure distribution (depending on whether the face is a target or a lure). Following previous work, we will assume that these target/lure distributions are Gaussian with means $\mu _{T,i}$/$\mu _{L,i}$ and variances $\sigma ^2_{T,i}$/$\sigma ^2_{L,i}$.^6^ It is further assumed that the recognition judgment regarding the *i*th face in a given sequence *s* is based on the comparison of their latent strength value with a response criterion $\tau _{0,i}$. Because a sequential lineup terminates as soon as the witness recognized a face, it follows that a “yes” response for *i*th face was preceded by *i* − 1 “no” responses.

For a given sequence *s* in a sequential lineup procedure, the probability of recognizing the face presented in position *i* corresponds to:

1 $$\begin{aligned} P({\texttt {"yes"}}, i, s) = \overbrace{\left( 1-\Phi \left( \frac{\tau _{0,i} - \mu _{s,i}}{\sigma _{s,i}}\right) \right) }^{\begin{array}{c} \texttt {probability\, of\, "yes"}\\ {\texttt {on\, position }} i \end{array}} \; \overbrace{\prod _{h < i}\Phi \left( \frac{\tau _{0,h} - \mu _{s,h}}{\sigma _{s,h}}\right) }^{\begin{array}{c} \texttt {probability\, of\, "no"}\\ {\texttt {in\, all\, previous\, positions}} \end{array}}, \end{aligned}$$

with $\Phi (\cdot )$ denoting the cumulative distribution function of the Standard Gaussian distribution. The subscripts [*s*, *i*] and [*s*, *h*] denote the latent strength distribution associated with the type of subject (target or lure) being presented in position *i* (or preceding position *h*) in a given sequence *s*.

In turn, the probability of rejecting all the faces in a given sequence *s* is given by:

2 $$\begin{aligned} P({\texttt{"no"}}, I, s) = \overbrace{\prod _{i=1}^I \Phi \left( \frac{\tau _{0,i} - \mu _{s,i}}{\sigma _{s,i}}\right) }^{\begin{array}{c} \texttt {probability\, of\, "no"}\\ {\texttt {for\, all\, positions}} \end{array}}. \end{aligned}$$

**Fig. 4:**
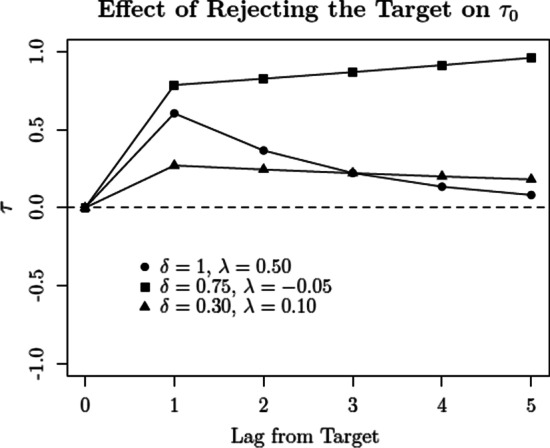
Effect of previously encountering and rejecting the target on $\tau _0$, as a function of parameters $\delta$ and $\lambda$. $\text {Lag} = 0$ denotes the value of $\tau$ when the target is encountered

**Fig. 5:**
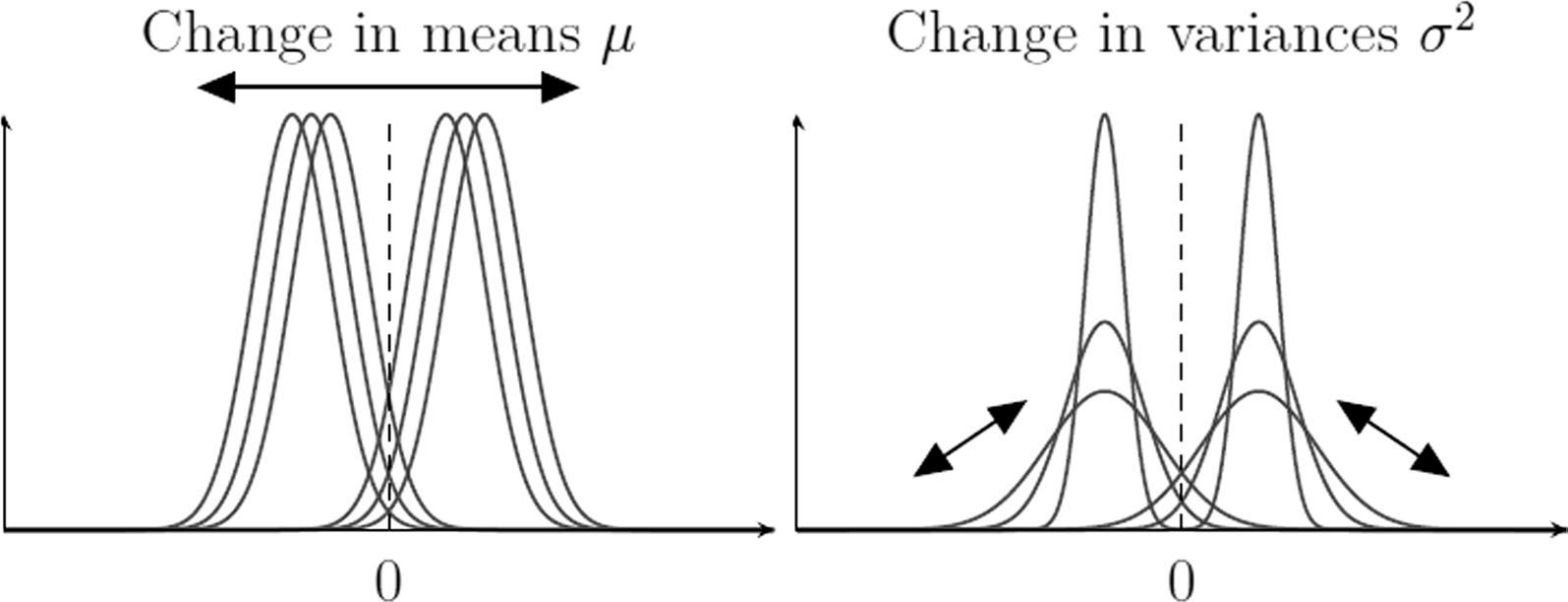
Illustration of how the latent strength distributions can change across sequence positions under different SDT models. The dashed lines indicate the origin of the latent strength scale

The SDT model specified by Eqs. 1 and 2 provides a general testbed for different hypotheses, which can be specified in terms of a hierarchy or grouping of models imposing different parameter restrictions (e.g., Batchelder & Riefer, 1990). In the present analysis, we considered the models listed in Table 2. These models result from a factorial combination of different hypotheses concerning response criteria and discriminability (for a similar approach, see van den Berg et al., 2014):

- **Response Criteria**
  $$\text {SDT}_{\varvec{\tau 1}}$$
(*fixed*): The same response criterion $\tau _0$ is used across all sequence positions, regardless of the position of the target in the sequence.
  $$\text {SDT}_{{\tau}2}$$
(*variation across sequence position*): Response criterion $\tau _0$ is free to vary across sequence position (e.g., position 1 versus position 5), but is invariant across different sequences (e.g., target absent vs. target in position 1). Note that the criterion is assumed to be unaffected by the type of faces previously encountered (i.e., whether or not the target was encountered before).
  $$\text {SDT}_{\varvec{\tau 3}}$$
(*variation due to target position*): Response criterion $\tau _0$ can vary as a function of encountering the target in a previous sequence position. Let $\tau _{0,i}$ be the response criterion for position *i* in a target-absent lineup. Now, let $\tau ^*_{0,i,h}$ be the criterion for position *i* in a sequence *s* in which the target was encountered in a previous position *h*, with $\tau ^*_{0,i,h} = \tau _{0,i} + \delta \exp (\lambda (h-i))$. This specification is able to capture the type of short-term and long-term effects considered by Treisman and Williams (1984), with parameter $\delta$ quantifying the “effect” of previously encountering the target, and $\lambda$ modulating its “decay” across the subsequent sequence positions.^7^ Figure 4 illustrates the relationship between parameter values and predictions. For example, when $\delta > 0$, the previous encounter of a target leads to an increase in the response criterion, which in turn favors the occurrence of “no” responses in subsequent sequence positions. Finally, note that it is being assumed that the response criterion $\tau _0$ is invariant for all lures prior to encountering the target (this follows from the assumption that lures are exchangeable).
  $$\text {SDT}_{\varvec{\tau 4}}$$
(*variation across sequence position and due to target position*): Response criterion $\tau _0$ can vary across sequence position (see $\text {SDT}_{\varvec{\tau 2}}$) *and* as a function of a previous encounter with the target (see $\text {SDT}_{\varvec{\tau 3}}$).
- **Discriminability**
  $$\text {SDT}_{\emptyset \;\;}$$
(*fixed discriminability*): Latent strength parameters of targets ($\mu _{T,i}$, $\sigma ^2_{T,i}$) and lures ($\mu _{L,i}$, $\sigma ^2_{L,i}$) are fixed across sequence positions.
  $$\text {SDT}_{\mu 1}$$
(*restricted change in means*): Across sequence positions, the means of the latent strength distributions for targets ($\mu _{T,i}$) and lures ($\mu _{L,i}$) can shift symmetrically around the origin of the scale (see Fig. 5). These symmetrical shifts are in line with the notion of “*differentiation*” that has been postulated by many prominent memory models (e.g., McClelland & Chappel, 1998; Shiffrin & Steyvers, 1997; for a review, see Criss & Koop, 2015). They can be achieved by introducing a factor $\alpha _i$, such that $\mu _{T,i} = \mu _T\alpha _i$ (with $\alpha _1 = 1$). In turn, symmetry is achieved by imposing the constraint that $\mu _{L,i} = - \mu _{T,i}$ (see Footnote 6). In this particular case, which follows Wilson et al. (2019), shifts in means are restricted to only take place once, right after sequence position 1. This is achieved by assuming that $\alpha _i = \alpha$, for $i>1$. Increases in discriminability, as postulated by the DFD hypothesis, are predicted when $\alpha >1$. Please note that the motivation behind the restriction that $\alpha _i = \alpha$, for $i>1$, is *purely pragmatic*; it is simply a way to capture a general pattern without having to introduce too many additional parameters.
  $$\text {SDT}_{\sigma 1}$$
(*restricted change in variances*): Across sequence positions, the variances of the latent strength distributions of targets ($\sigma ^2_{T,i}$) and lures ($\sigma ^2_{L,i}$) can change by a common factor $\xi _i$, such that $\sigma ^2_{T,i} = \xi _i\cdot \sigma ^2_{T}$ and $\sigma ^2_{L,i} = \xi _i\cdot \sigma ^2_{L}$, with $\xi _1 = 1$ (see Fig. 5). This change captures the kinds of increase/decrease in “noise” that have been postulated in the literature (e.g., Criss et al., 2011; Osth et al., 2018; Wixted & Mickes, 2014). Similar to $\text {SDT}_{\mu 1}$, changes in variances are restricted to only take place right after sequence position 1. This is achieved by assuming that $\xi _i = \xi$, for $i>1$. Increases in discriminability, as postulated by the DFD hypothesis, are predicted when $0<\xi <1$. As in the previous case, the restriction that $\xi _i = \xi$, for $i>1$, is an attempt to avoid models with an excessive number of free parameters.
  $$\text {SDT}_{\mu 2}$$
(*unrestricted change in means*): Similar to $\text {SDT}_{\mu 1}$, the difference being that mean shifts can take place across all sequence positions.
  $$\text {SDT}_{\sigma 2}$$
(*unrestricted change in variances*): Similar to $\text {SDT}_{\sigma 1}$, the difference being that changes in variance can take place across all sequence positions.

The factorial combination of these different assumptions results in $4\times 5 = 20$ candidate models. One advantage of this factorial approach is that it allows us to check whether or not the support for a given hypothesis is dependent on the other hypotheses being considered (for a discussion, see van den Berg et al., 2014).

Table 2 lists all the twenty models along with their performance in terms of badness of fit (quantified by the $G^2$ statistic) as well as in terms of the Akaike and Bayesian information criteria (AIC and BIC, respectively), which penalize models for their flexibility using their respective numbers of free parameters as a proxy for the latter (Myung & Pitt, 2004). Altogether, we see two clear results: First, there is very strong support for the hypothesis that response criteria vary as a function of sequence position as well as target rejection. In comparison with the most restricted model, SDT$_{\tau 1,\,0}$, badness of fit is drastically reduced when allowing response criteria to vary (SDT$_{\tau 4,\,0}$). In contrast, when only allowing discriminability to vary across sequence positions, we obtained a much more modest reduction (SDT$_{\tau 1,\,\mu 2}$).

**Fig. 6:**
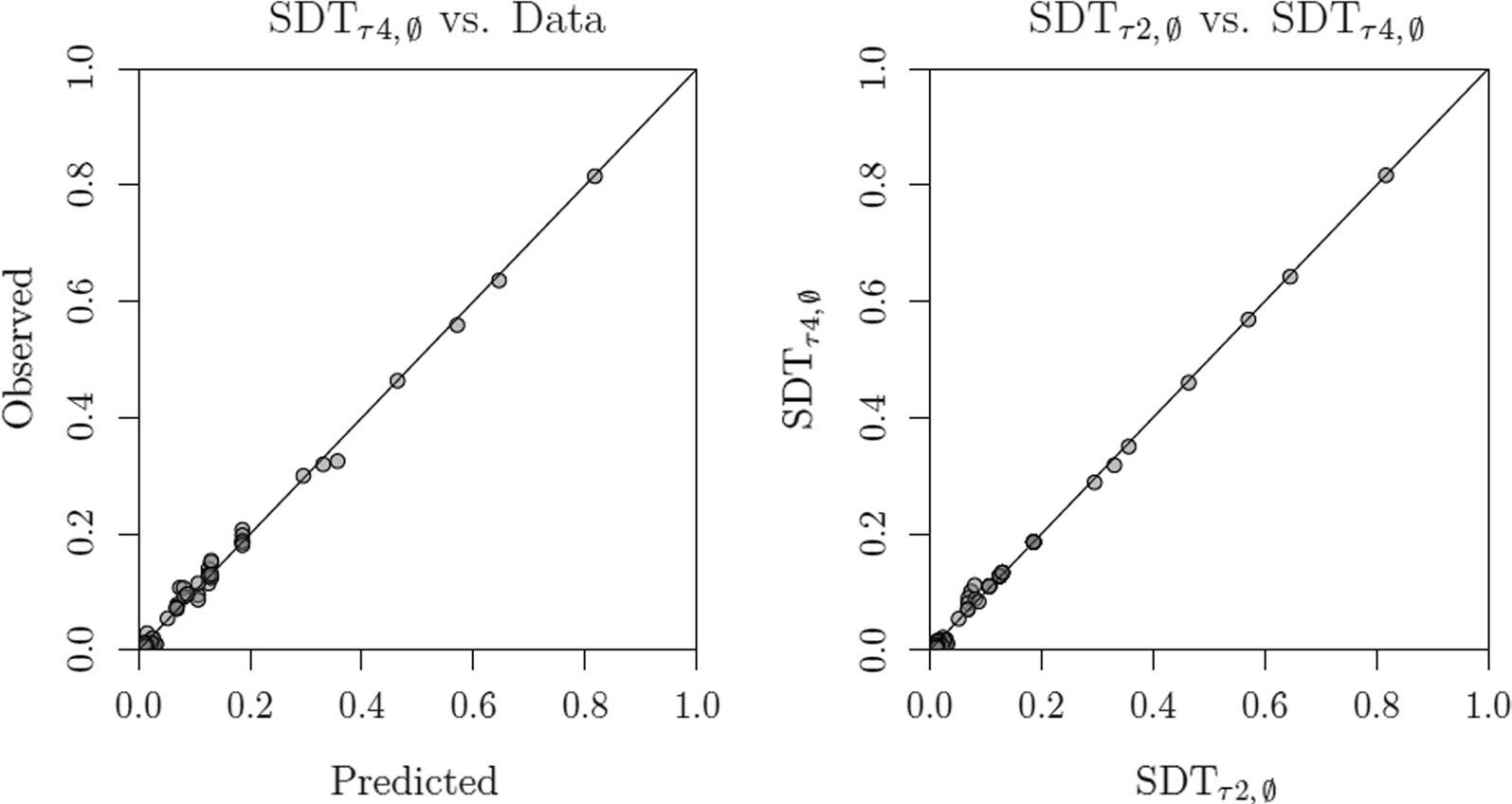
Left panel: Comparison of model predictions (SDT$_{\tau 4,\,\emptyset }$) and the data. Right panel: Comparison of model predictions coming from the SDT$_{\tau 2,\,\emptyset }$ and SDT$_{\tau 4,\,\emptyset }$ models

**Fig. 7:**
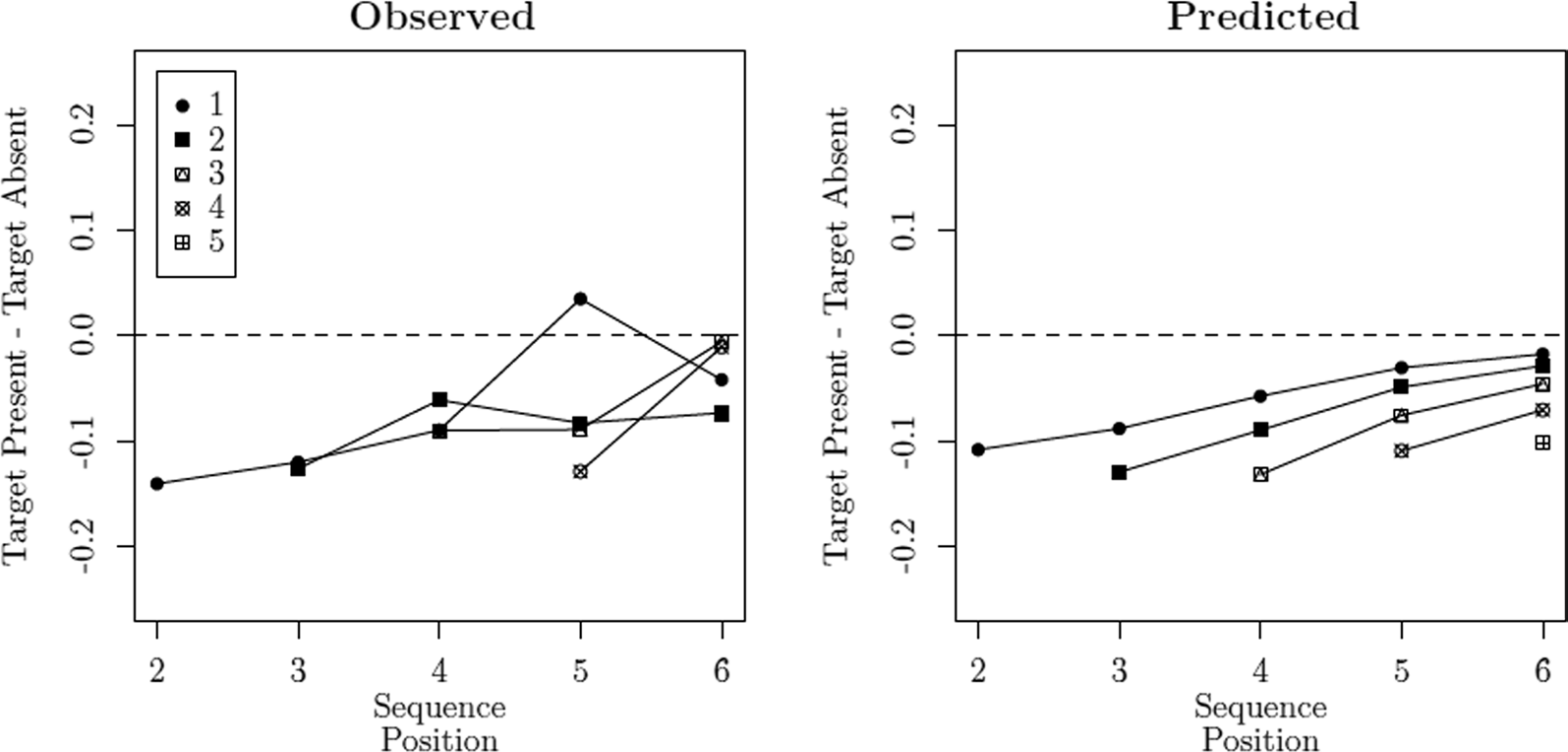
Differences in the conditional probability of responding “yes” to a lure in sequence position *i* between target-present lineups (in which the target was previously rejected) and target-absent lineups. The different symbols/lines distinguish the preceding position taken by the target in target-present lineups (reanalysis of Wilson et al., 2019)

These modeling results are corroborated by the left panel of Fig. 6, where we contrast the predictions of the best-performing model, SDT$_{\tau 4,\,\emptyset }$, with the observed choice proportions. They are also corroborated by Fig. 7, which contrasts the conditional probabilities of recognizing a lure on sequence position *i* in target-absent lineups and target-present lineups when the target was previously rejected: The differences found in the data (Fig. 7, Left Panel) show that the probability of recognizing a lure is lower when the target was previously rejected, when compared to the lure rejection rates found in target-absent lineups (for similar results, see Dunn et al., 2022). We also see that this difference tends to decrease across sequence positions. This general pattern is captured by the predictions of SDT$_{\tau 4,\,\emptyset }$ (Fig. 7, Right Panel).^8^ Aside from its relative performance, the misfits produced by model SDT$_{\tau 4,\,\emptyset }$ were not statistically significant ($p = .08$). This result is particularly impressive given the large sample size.

**Table 2:** Model selection results (Reanalysis of Wilson et al., 2019)

Binary judgments				
Model	Parameters	$G^2$	AIC	BIC
SDT$_{\tau 1,\,\emptyset }$	3	187	193	214
SDT$_{\tau 1,\,\mu 1}$	4	182	190	218
SDT$_{\tau 1,\,\sigma 1}$	4	182	190	218
SDT$_{\tau 1,\,\mu 2}$	8	152	168	225
SDT$_{\tau 1,\,\sigma 2}$	8	150	166	222
SDT$_{\tau 2,\,\emptyset }$	8	122	138	194
SDT$_{\tau 2,\,\mu 1}$	9	120	138	202
SDT$_{\tau 2,\,\sigma 1}$	9	120	138	202
SDT$_{\tau 2,\,\mu 2}$	13	118	144	236
SDT$_{\tau 2,\,\sigma 2}$	13	118	144	236
SDT$_{\tau 3,\,\emptyset }$	5	106	116	151
SDT$_{\tau 3,\,\mu 1}$	6	104	116	159
SDT$_{\tau 3,\,\sigma 1}$	6	104	116	159
SDT$_{\tau 3,\,\mu 2}$	10	72	92	163
SDT$_{\tau 3,\,\sigma 2}$	10	70	90	160
SDT$_{\tau 4,\,\emptyset }$	10	44	**64**	**134**
SDT$_{\tau 4,\,\mu 1}$	11	43	65	143
SDT$_{\tau 4,\,\sigma 1}$	11	43	65	143
SDT$_{\tau 4,\,\mu 2}$	15	40	70	175
SDT$_{\tau 4,\,\sigma 2}$	15	39	69	175

*Confidence rating judgments*				
SDT$_{\tau 4,\,\emptyset }$	30	177	**237**	**448**
SDT$_{\tau 4,\,\mu 1}$	31	177	239	457
SDT$_{\tau 4,\,\sigma 1}$	31	177	239	457
SDT$_{\tau 4,\,\mu 2}$	36	174	246	499
SDT$_{\tau 4,\,\sigma 2}$	36	173	245	498

The best-performing values according to AIC and BIC are underlined and in bold font

The second main result is that the support for a change in discriminability seems to hinge on the constraints imposed at the level of response criteria: *Support is only found when the response criteria are fixed*. For example, compare the performance differences between SDT$_{\tau 1,\,\emptyset }$ and SDT$_{\tau 1,\,\mu 1}$, and between SDT$_{\tau 4,\,\emptyset }$ and SDT$_{\tau 4,\,\mu 1}$. This result echoes previous modeling reports showing that the testing of specific components (e.g., discriminability) can be severely biased by the auxiliary constraints being imposed elsewhere (e.g., Barr et al., 2013; Kellen et al., 2013b), especially when these constraints are known to increase misfits by a non-negligible amount (see Maydeu-Olivares & Cai, 2006). This aspect is relevant when considering the fact that the present lack of support for differences in discriminability runs counter to Wilson et al.’s (2019) results with the same data. Aside from the fact that the data were structured differently and that they considered confidence ratings from the onset, we note that Wilson et al.’s main result assumed that response criteria were either invariant across sequence positions or allowed to vary in a highly constrained manner (namely, in the form of lockstep shifts).

The parameter estimates obtained with the three best-performing models (according to AIC and BIC) are reported in Table 3. A major discrepancy between the models can be found at the level of the $\sigma ^2_T$ estimates: The $\sigma ^2_T$ estimate obtained with the SDT$_{\tau 4,\,0}$ model is very close to 1, which is consistent with previous reports (Kaesler et al., 2020). In contrast, the SDT$_{\tau 4,\,\mu 1}$ and SDT$_{\tau 4,\,\sigma 1}$ models yielded much lower estimates, closer to the results originally reported by Wilson et al. (2019). We evaluated these parameter estimates by fitting constrained versions of their respective models in which $\sigma ^2_T = \sigma ^2_L = 1$. In all cases, the restriction led to negligible differences in misfit (e.g., for SDT$_{\tau 4,\,\mu 1}$, $\Delta G_{\text {df}=1}^2 = 0.29$, $p = .29$). These results show that it is very difficult to reliably estimate $\sigma ^2_T$ with binary judgments, despite the large sample size and the many degrees of freedom provided by the data. This situation raises the possibility that the present lack of support for the DFD hypothesis is due to our reliance on binary judgments. We will address this possibility later on.

**Table 3:** Parameter estimates for models SDT$_{\tau 4,\, \emptyset }$, SDT$_{\tau 4,\, \mu 1}$, and SDT$_{\tau 4,\, \sigma 1}$ (reanalysis of Wilson et al., 2019)

Binary judgments					
SDT$_{\tau 4,\, \emptyset }$		SDT$_{\tau 4,\, \mu 1}$		SDT$_{\tau 4,\, \sigma 1}$	
$\mu _T$	0.91	$\mu _{T}$	0.66	$\mu _{T}$	0.66
$\sigma ^2_T$	1.03	$\sigma ^2_{T}$	0.21	$\sigma ^2_{T}$	0.21
$\tau _{0,1}$	− 0.01	$\alpha _{1}$	1	$\xi _{1}$	1
$\tau _{0,2}$	0.09	$\alpha _{2-6}$	1.17	$\xi _{2-6}$	0.73
$\tau _{0,3}$	− 0.07	$\tau _{0,1}$	0.24	$\tau _{0,1}$	0.24
$\tau _{0,4}$	− 0.09	$\tau _{0,2}$	0.23	$\tau _{0,2}$	0.19
$\tau _{0,5}$	0.06	$\tau _{0,3}$	0.08	$\tau _{0,3}$	0.07
$\tau _{0,6}$	0.11	$\tau _{0,4}$	0.06	$\tau _{0,4}$	0.05
$\delta$	1.07	$\tau _{0,5}$	0.21	$\tau _{0,5}$	0.18
$\lambda$	0.50	$\tau _{0,6}$	0.25	$\tau _{0,6}$	0.22
		$\delta$	1.07	$\delta$	0.91
		$\lambda$	0.50	$\lambda$	0.50

Confidence rating judgments					
SDT$_{\tau 4,\, \emptyset }$		SDT$_{\tau 4,\, \mu 1}$		SDT$_{\tau 4,\, \sigma 1}$	
$\mu _T$	0.77	$\mu _{T}$	0.76	$\mu _{T}$	0.76
$\sigma ^2_T$	0.51	$\sigma ^2_{T}$	0.51	$\sigma ^2_{T}$	0.51
$\tau _{0,1}$	0.13	$\alpha _{1}$	1	$\xi _{1}$	1
$\tau _{0,2}$	0.22	$\alpha _{2-6}$	1.02	$\xi _{2-6}$	0.97
$\tau _{0,3}$	0.08	$\tau _{0,1}$	0.13	$\tau _{0,1}$	0.13
$\tau _{0,4}$	0.05	$\tau _{0,2}$	0.21	$\tau _{0,2}$	0.21
$\tau _{0,5}$	0.20	$\tau _{0,3}$	0.08	$\tau _{0,3}$	0.07
$\tau _{0,6}$	0.25	$\tau _{0,4}$	0.05	$\tau _{0,4}$	0.05
$\delta$	1.04	$\tau _{0,5}$	0.20	$\tau _{0,5}$	0.19
$\lambda$	0.49	$\tau _{0,6}$	0.25	$\tau _{0,6}$	0.24
$\omega$	1.44	$\delta$	1.05	$\delta$	1.03
$\eta$	− 0.03	$\lambda$	0.49	$\lambda$	0.49
$\gamma$	0.12	$\omega$	1.37	$\omega$	1.36
		$\eta$	− 0.04	$\eta$	− 0.04
		$\gamma$	0.11	$\gamma$	0.10

Moreover, the visual inspection of the response criteria estimates suggests that they vary in a non-monotonic fashion across sequence positions. Forcing $\tau _0$ to be fixed across sequence positions invariably led to large, statistically significant increases in badness of fit (smallest $\Delta G_{\text {df}=6}^2 = 30.88$, largest $p < .001$). In order to test the robustness of the non-monotonic pattern found, we evaluated the hypothesis that the response criteria are *monotonically increasing* across sequence positions, such that $\tau _{0,h} \le \tau _{0,i}$ for all $h < i$.^9^ This hypothesis was motivated by previous work suggesting that the probability of a “no” response is greater when preceded by another “no” response (e.g., Malmberg & Annis, 2012), along with the fact that by design, a typical sequential lineup judgment at position *i* implies the rejection of all preceding faces.^10^ The difference in badness of fit between SDT$_{\tau 4,\,\emptyset }$ and a restricted version enforcing *monotonically increasing* criteria was considerable ($\Delta G^2 = 38.58$, $p < .001$).^11^ We also tested the alternative hypothesis of *monotonically decreasing* criteria, a restriction that also led to statistically significant increases in misfit ($\Delta G^2 = 48.35$, $p < .001$). Altogether, these results suggest that the non-monotonic pattern found in the response criteria estimates cannot be dismissed as the mere outcome of sampling variability.

Regarding the effect of target rejections on response criteria, the $\lambda$ estimates obtained across the three best-performing models suggest that this effect decays as one progresses through the sequential lineup. This result is consistent with pattern observed in Fig. 7, where we see a diminishing decrease in “yes” responses for lures, when comparing target-present lineups (in which the target was previously rejected) and target-absent lineups. We tested this “decay hypothesis” by comparing the SDT$_{\tau 4,\,0}$ model with a restricted version in which $\lambda = 0$. The increase in misfit was considerable ($\Delta G_{\text {df}=1}^2 = 15.82$, $p < .001$), which corroborates the notion of a decaying effect.

Lastly, a methodological point regarding the overall evaluation of models: Although SDT$_{\tau 4,\,\emptyset }$ clearly outperforms its competitors, this difference is barely visible when inspecting their predicted response probabilities. Consider the comparison between SDT$_{\tau 2,\,\emptyset }$ and SDT$_{\tau 4,\,\emptyset }$, which differ dramatically in terms of badness of fit. As shown on the right panel of Fig. 6, their predictions barely differ. These differences only become clear (at least visually) when conditioning each response probability on the preceding responses, as shown in Fig. 7, in which models such as the SDT$_{\tau 2,\,\emptyset }$ predict a *constant zero difference*. It should be noted that the conditional response probabilities being contrasted in Fig. 7 are *discrete hazard functions* (Chechile, 2003). One of the attractive features of hazard functions is that they often reveal differences between models that can be virtually indistinguishable otherwise (e.g., Chechile, 2006). The present case is another demonstration of their usefulness.

### Analysis of confidence ratings

Although confidence judgments are not necessary for SDT parameters to be reliably estimated (e.g., Dube & Rotello, 2012; Kellen & Klauer, 2011), it is clear that this is not the case with sequential lineups. Despite the large sample size and the many degrees of freedom, the binary judgments in Wilson et al.’s (2019) data are yielding unreliable $\sigma ^2_T$ estimates. This unfortunate situation is likely to have affected our evaluation of the DFD hypothesis. In response, we extended our best-performing models so that they can accommodate confidence rating judgments.^12^ This is extension is achieved by introducing an ordered set of confidence criteria $\tau _k$ that take on more extreme values than the criterion $\tau _0$ responsible for the binary judgments (see the top panel of Fig. 1). As previously discussed, the sequential lineup procedure yields a *family* of confidence rating ROCs, each of them characterizing one of the guilty suspect sequence positions (see Fig. 3). The two studies reported by Wilson et al. (2019) requested participants to express their confidence on a slider ranging from $-100$ to 100. In line with previous studies, we only considered the confidence ratings for “yes” responses (e.g., Juslin et al., 1996; Mickes, 2015; Wixted et al., 2015), which we divided into four equally spaced intervals (0–25, 26–50, 51–75, and 76–100). In light of the previous results showing that the response criterion changes across sequence positions, all of the models considered here will allow confidence criteria to vary freely across sequence positions (but see Footnote 12).

We decided to extend our models so that we could evaluate the effect of target rejection at the level of confidence judgments. For $K+1$ levels of confidence, with $k = 1, \dots , K$, let:

$$\begin{aligned} \tau _{k,i} = \tau _{0,i} + \sum _{m=1}^k \kappa _{m,i} \end{aligned}$$

denote confidence criteria, with $\kappa _{i,m}$ being nonnegative increments over $\tau _i$. Confidence criteria $\tau _{i,k}$ are applied to the *i*th sequence position of target-absent lineups or target-present lineups up to the encountering the target.

In the case of target-present lineups in which the target was previously encountered in sequence position *h*, the confidence criteria for sequence position *i* are specified as follows:

$$\begin{aligned} \tau ^*_{k,i,h} = \tau ^*_{0,i,h} + \sum _{m=1}^{k} \kappa ^*_{m,i,h}, \end{aligned}$$

with

$$\begin{aligned} \kappa ^*_{m,i,h}&= \kappa _{m,i}\cdot \rho _{i,h}\exp (-\gamma \cdot m),\\ \rho _{i,h}&= \omega \exp (\eta (h-i)). \end{aligned}$$

**Fig. 8:**
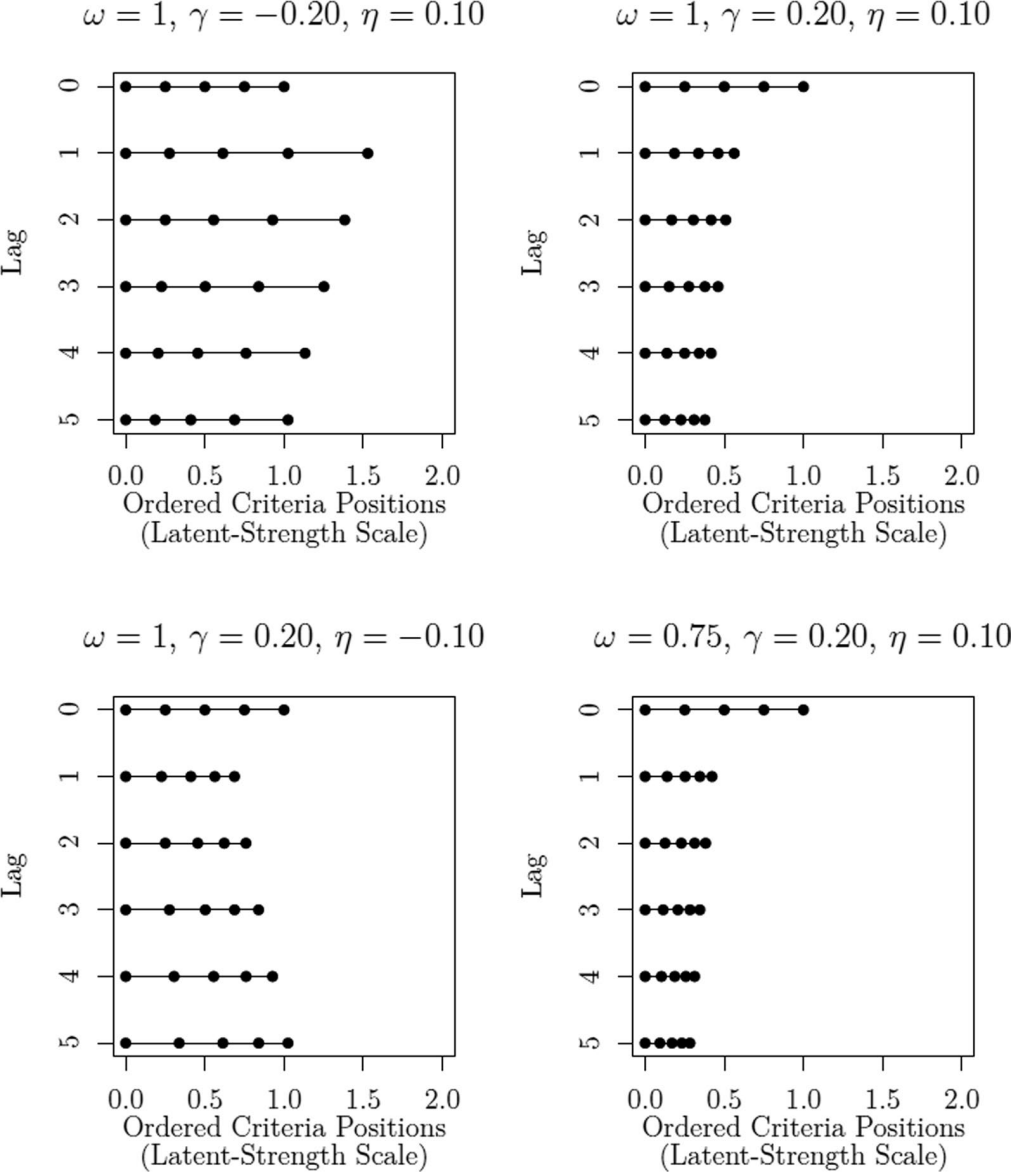
Examples of the effect of previously encountering and rejecting the target on ordered confidence criteria $\tau _i$, which is established as a function of parameters $\omega$, $\gamma$, and $\eta$ and Lag $= i-h$. $\text {Lag} = 0$ denotes the value of the criteria when the target is encountered. For illustrative purposes, $\tau _0$ remains fixed at 0 across lags and the contiguous confidence criteria in $\text {Lag} = 0$ are equidistant

According to this parameterization, increments $\kappa _{i,m}$ can increase or decrease as a function of $\rho _{i,h} \ge 0$. In turn, $\rho _{i,h}$ is defined as a function of an “effect” parameter $\omega$ and a “decay” parameter $\eta$, similar to the way we specified $\tau ^*_{i,h}$ as a function of $\delta$ and $\lambda$ (see Fig. 4). Finally, parameter $\gamma$ determines how the magnitude of the effects changes across confidence levels. When $\gamma > 0$, the effect decreases as a function of the confidence level; when $\gamma < 0$ it decreases. Figure 8 illustrates how the criteria are affected by the previous encounter of the target, as a function of $\omega$, $\eta$, and $\gamma$. The motivation behind this parameterization is that it provides a convenient way to investigate a number of effects while sidestepping the need for an unreasonably large number of parameters (for a similar approach, see Selker et al., 2019). Relative to the model specifications used in the case of binary judgments, the total number of additional parameters is $6\times (K-1) + 3$, with the first term corresponding to the total number of increments $\kappa _{i,m}$ and the second term to parameters $\omega$, $\eta$, and $\gamma$. If we instead decided to specify completely unconstrained confidence criteria, we would need to introduce $21\times (K-1)$ parameters.

**Fig. 9:**
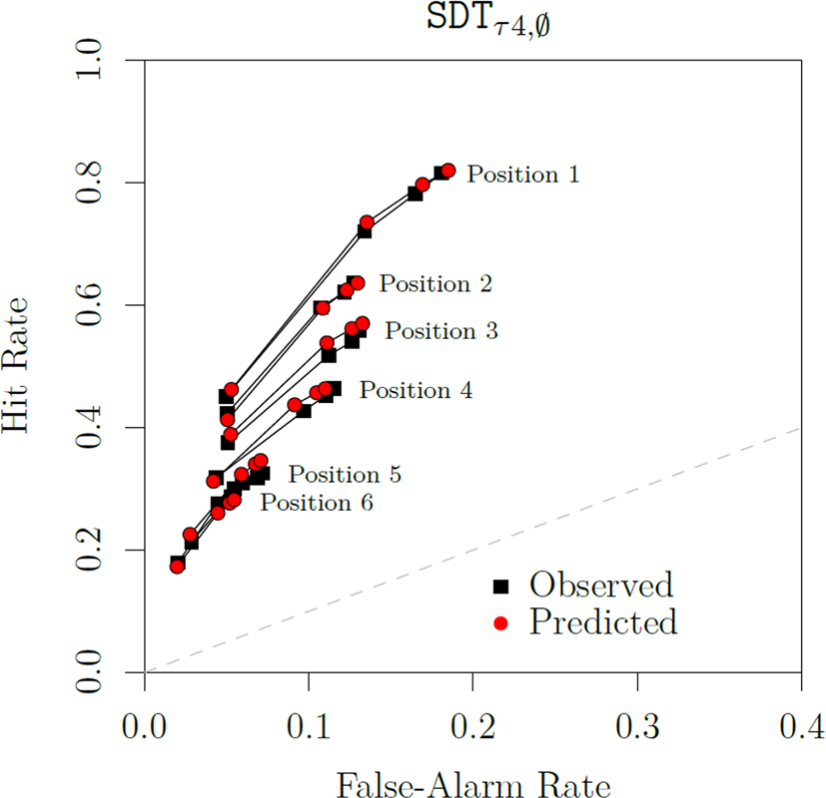
Observed confidence rating ROCs and the corresponding predictions from SDT$_{\tau 4, \emptyset }$ (Reanalysis of Wilson et al., 2019)

The model-fitting results are reported in Tables 2 and 3. Figure 9 contrasts the predictions of the best-performing model, SDT$_{\tau 4,\,\emptyset }$. Based on a visual inspection, the predicted confidence rating ROCs are close to the observed ones.^13^ Although model misfits were found to be statistically significant ($p = .01$), the observed badness of fit is not completely unacceptable when taking into consideration the large sample size (see Bröder & Schütz, 2009).^14^ The other candidate models considered, which include SDT$_{\tau 4,\,\emptyset }$ as a special case, only produced marginal improvements over the latter (largest $\Delta G^2 = 4.4$, $p = .49$). This result is reflected in the $\alpha$ and $\xi$ estimates reported in Table 3, which barely deviated from 1. These results corroborate the previous analysis of binary judgments in which we failed to accrue any support for the DFD hypothesis.

In the present analysis of confidence ratings, imposing the equal variance restriction $\sigma ^2_T = \sigma ^2_L = 1$ on SDT$_{\tau 4,\,\emptyset }$ led to large increases in badness of fit ($\Delta G_{\text {df}=1}^2 = 93.51$, $p<.0001$), which indicates that the $\sigma ^2_T$ estimates are reliably below 1. This result, which is in line with previous reports on lineup procedures (e.g., Wixted et al., 2018), shows that confidence ratings play a critical role in estimating $\sigma ^2_T$ in the context of sequential lineups. They are more than a convenient way of augmenting the degrees of freedom provided by the data.

Finally, we evaluated the impact of allowing for confidence criteria to be affected by a previous target rejection. Across all the models considered, the restriction of $\omega$, $\eta$, and $\gamma$ had a minimal impact (largest $\Delta G_{\text {df}=3}^2 = 1.11$, smallest $p = .77$). Given these results, it seems unwise to try to extract implications from the estimates of $\omega$, $\eta$, and $\gamma$ reported in Table 3. An obvious culprit here is the fact that the number of cases in which witnesses miss the target and subsequently accept a lure is quite small (see the italic frequencies in Table 1). Not surprisingly, this issue becomes increasingly acute when “unpacking” these already uncommon responses in terms of confidence levels.

### The question of unconstrained confidence criteria

One concern with the results above is that (1) allowing confidence criteria to be freely estimated introduces an amount of flexibility that is unwarranted, and (2) this flexibility is limiting the ability to detect changes in discriminability. We will address (1) here and turn to (2) in the subsection below. Let us consider the SDT$_{\tau 4,\,\emptyset }$ and SDT$_{\tau 4,\,\mu 1}$ models: If we constrained the $\kappa _{m,i}$ parameters to be the same across positions in both models, then their difference in badness of fit becomes statistically significant; $\Delta G_{\text {df} = 1}^2 = 7.45$, $p = .008$, a result that supports the DFD hypothesis. However, these criteria-constrained models fit the data much worse than their unconstrained counterparts (smallest $\Delta G_{\text {df} = 15}^2 = 98$, $p < .0001$). What these results show is that, similar to our previous analysis of binary judgments, *support for the DFD hypothesis is apparently only found when imposing constraints that are rejected by the data*. In terms of AIC and BIC, there is a disagreement: AIC supports the models with unconstrained confidence criteria (in line with the null hypothesis tests), whereas BIC supports their constrained counterparts. However, it is well known that BIC can impose unreasonably large penalties *per additional parameter* (see Kellen et al., 2013a) and end up supporting models that fail to provide an acceptable fit of the data, in detriment of models that do (see Gelman & Rubin, 1995).

In reaction, one could argue that the assumption that response criteria are fixed across sequence positions is *more principled* than the alternative of allowing them to vary haphazardly, regardless of badness-of-fit or model selection results. We identify a number of problems with this stance: First, it ignores or downplays the fact that we are dealing with *aggregate data coming from heterogeneous respondents*. There is a large body of work showing how certain features found in aggregate data might not be representative of any of the individual respondents (e.g., Regenwetter & Robinson, 2017; Regenwetter et al., in press; but see Kellen, in press). In other words, data aggregation might turn perfectly sensible patterns among individual respondents into something completely different, perhaps something disordered or incoherent. Second, the solution proposed—restricting response criteria across sequence positions—is not well supported on methodological nor conceptual grounds: On the one hand, the proposed restriction runs counter to the well-established practice of making models *more flexible* in order to deal with individual heterogeneity (see Barr et al., 2013; Regenwetter & Robinson, 2017). On the other hand, it invites the researcher to entertain a scenario in which changes in response criteria across sequence position are precluded *a priori* while allowing criteria to change as a function of a previous encounter with the target. Third and finally, the very notion that fixing response criteria across positions constitutes a principled move is likely predicated on an analogical overreach: The fact that response criteria are typically assumed to be fixed across recognition memory trials does not underwrite its enforcement in other domains such as eyewitness identification, regardless of the fact that its data can be understood in similar ways (e.g., in terms of ROC functions) and successfully characterized by the same model framework. One should not overlook the possibility of perceiving sequential lineups through a different lens that finds criterion changes highly plausible; e.g., casting sequential lineups as an “optional-stopping problem” (see Baumann et al., 2020; Lee & Courey, 2021).

### Statistical power when evaluating the DFD hypothesis

We investigated the possibility that the failure to find evidence in support of the DFD hypothesis is due to a lack of statistical power. Simply put, it is possible that the flexibility gained from letting response criteria be completely unconstrained is masking actual differences in discriminability (for an example of how the flexibility of response criteria can affect statistical power when testing SDT models, see Kellen et al., 2013b). Such a scenario would explain the discrepant results found when modeling binary judgments, between the comparison of SDT$_{\tau 1,\,\emptyset }$ with SDT$_{\tau 1,\,\mu 1}$ (which supported a difference in discriminability) and the comparison between SDT$_{\tau 4,\,\emptyset }$ and SDT$_{\tau 4,\,\mu 1}$ (which did not). This scenario would also speak against our exclusive use of models with unconstrained criteria when modeling confidence ratings and ultimately vindicate the “restrictive stance” criticized in the previous section.

**Fig. 10:**
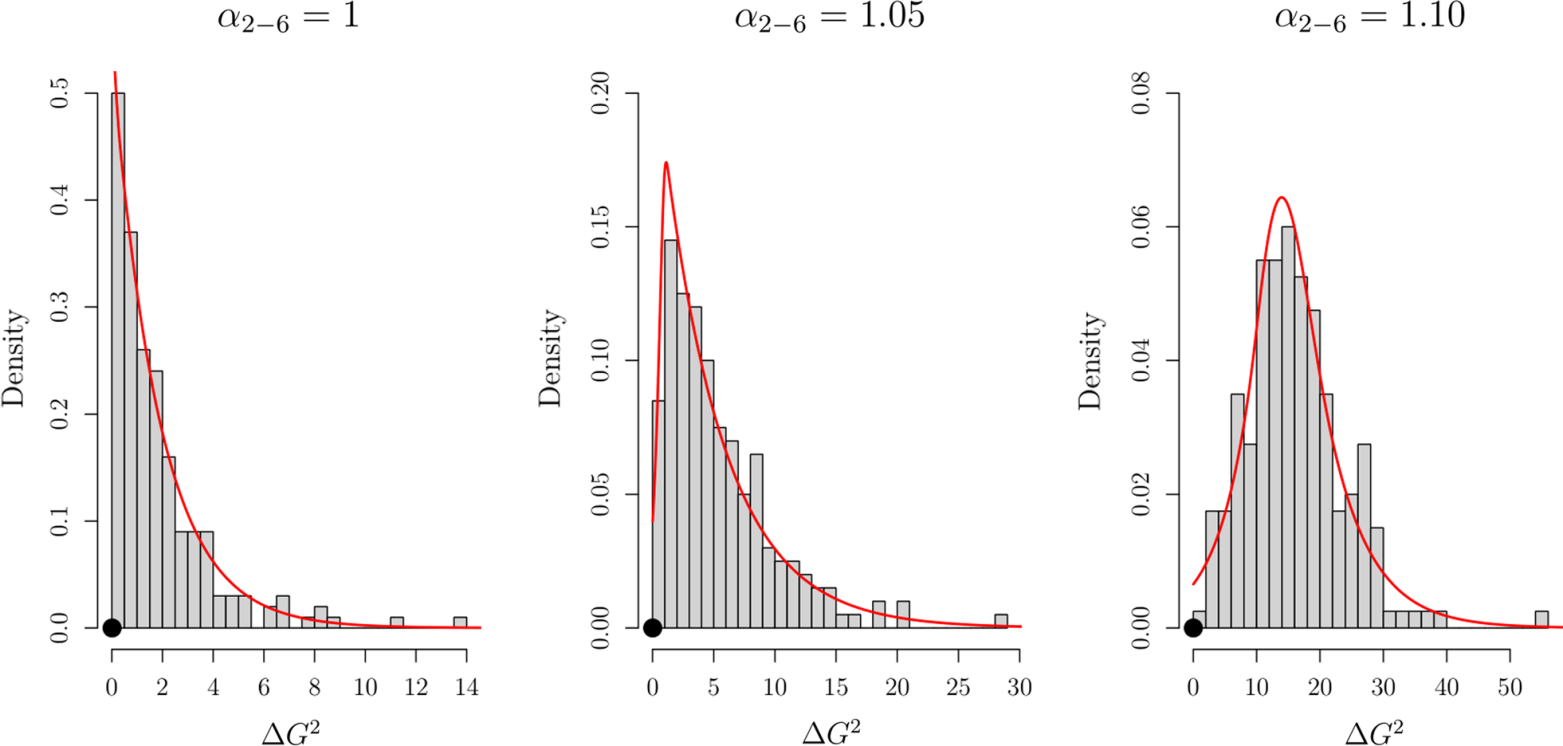
Distribution of $\Delta G^2$ values, obtained from the comparison of the SDT$_{\tau 4,\,\emptyset }$ and SDT$_{\tau 4,\,\mu 1}$ models, when generating the confidence rating data from the latter under different values of $\alpha _{2-6}$. Two-hundred datasets were generated per scenario. The black circle corresponds to the $\Delta G^2 = 0.01$ observed with the real data (see Table 3). The red lines correspond to polynomial spline approximations of the $\Delta G^2$ distributions. Please note that the ranges of both axes vary across panels

In order to evaluate this possibility, we simulated confidence rating data using the parameter estimates obtained with the SDT$_{\tau 4,\,\mu 1}$ (see the bottom half of Table 2), with the exception of parameters $\alpha _{2-6}$ which were fixed to 1, 1.05, or 1.10. Under these parameter estimates, the difference between $\mu _T$ and $\mu _L$ is expected to increase by 0%, 5%, or 10% between sequence position 1 and positions 2-6. We then used the SDT$_{\tau 4,\,\emptyset }$ and SDT$_{\tau 4,\,\mu 1}$ models to fit these data and evaluated their difference in badness of fit (quantified by $\Delta G^2$). We repeated this entire procedure 200 times for each of the $\alpha _{2-6}$ values considered. Figure 10 illustrates the distribution of $\Delta G^2$ values obtained for each value of $\alpha _{2-6}$ considered, along with the $\Delta G^2$ value that was obtained when modeling the real data (solid circle). The distribution of $\Delta G^2$ values in the case of $\alpha _{2-6} = 1$, which corresponds to the “null hypothesis”, is somewhat similar to the asymptotic $\chi ^2_{\text {df} = 1}$ distribution, although it takes on more extreme results with greater frequency: The proportion of statistically significant cases ($p < .05$) was 12% instead of the nominal 5%. In the other two cases, $\alpha _{2-6} = 1.05$ and 1.10, the obtained $\Delta G^2$ values were deemed statistically significant in 54% and 97% of the times, respectively.

Beyond binary proclamations of “statistical significance,” the distributions illustrated in Fig. 10 show that the $\Delta G^2 = 0.01$ obtained with the real data is largely consistent with the distribution obtained under the null hypothesis and inconsistent with the distributions obtained under the two alternative hypotheses considered: When using polynomial splines to estimate the probability densities of each $\Delta G^2$ distribution (see Fig. 10), we find that the observed difference of 0.01 is *fourteen* and *eighty-seven times* more likely under the null hypothesis $\alpha _{2-6} = 1$ than under the alternatives $\alpha _{2-6} = 1.05$ and 1.10, respectively. These results do not change much when considering $\Delta G^2$ values ranging between 0 and 1.5: The null is still *three* and *forty-nine* times more likely than the two alternatives. Overall, these simulation results produce a very clear message: The ability to detect moderate differences in discriminability (e.g., a 10% increase) is quite high, even when response criteria are allowed to vary across sequence positions, which means that there is no statistical power or likelihood ratio rationale for questioning the specification of unconstrained response criteria.

### Looking back at the validity of binary response modeling outcomes

Our analyses of binary and confidence judgments from Wilson et al. (2019) showed how the latter are necessary in order to estimate $\mu _T$ and $\sigma ^2_T$ with reasonable precision. This result shows that—for purposes of parameter estimation—binary judgments in sequential lineups have limited diagnostic value despite the large number of degrees of freedom that they provide. *Confidence ratings turn out to be necessary*.

At first glance, one might see this result as a mere demonstration of something that is well established in the context of recognition memory: that binary responses alone (i.e., without response bias manipulations) cannot support a complete SDT characterization, an inability that ultimately leads to failures in distinguishing differences in discriminability from differences in response criteria (see Rotello et al., 2008; Verde et al., 2006). One problem with this view is that it risks overlooking the consistency between the model-comparison results obtained with binary and confidence judgments (see Table 2). In fact, if we use the $\mu _T$ and $\sigma ^2_T$ estimates in Table 3 to compute the discriminability measure $d_a$, we end up with very similar values:^15^ 1.79, 1.72, and 1.72, in the case of binary judgments, and 1.80, 1.75, and 1.75, in the case of confidence ratings. This consistency suggests that binary judgments from sequential lineups might still be informative in specific circumstances. In order to identify these circumstances, we conducted a number of simulations.

**Fig. 11:**
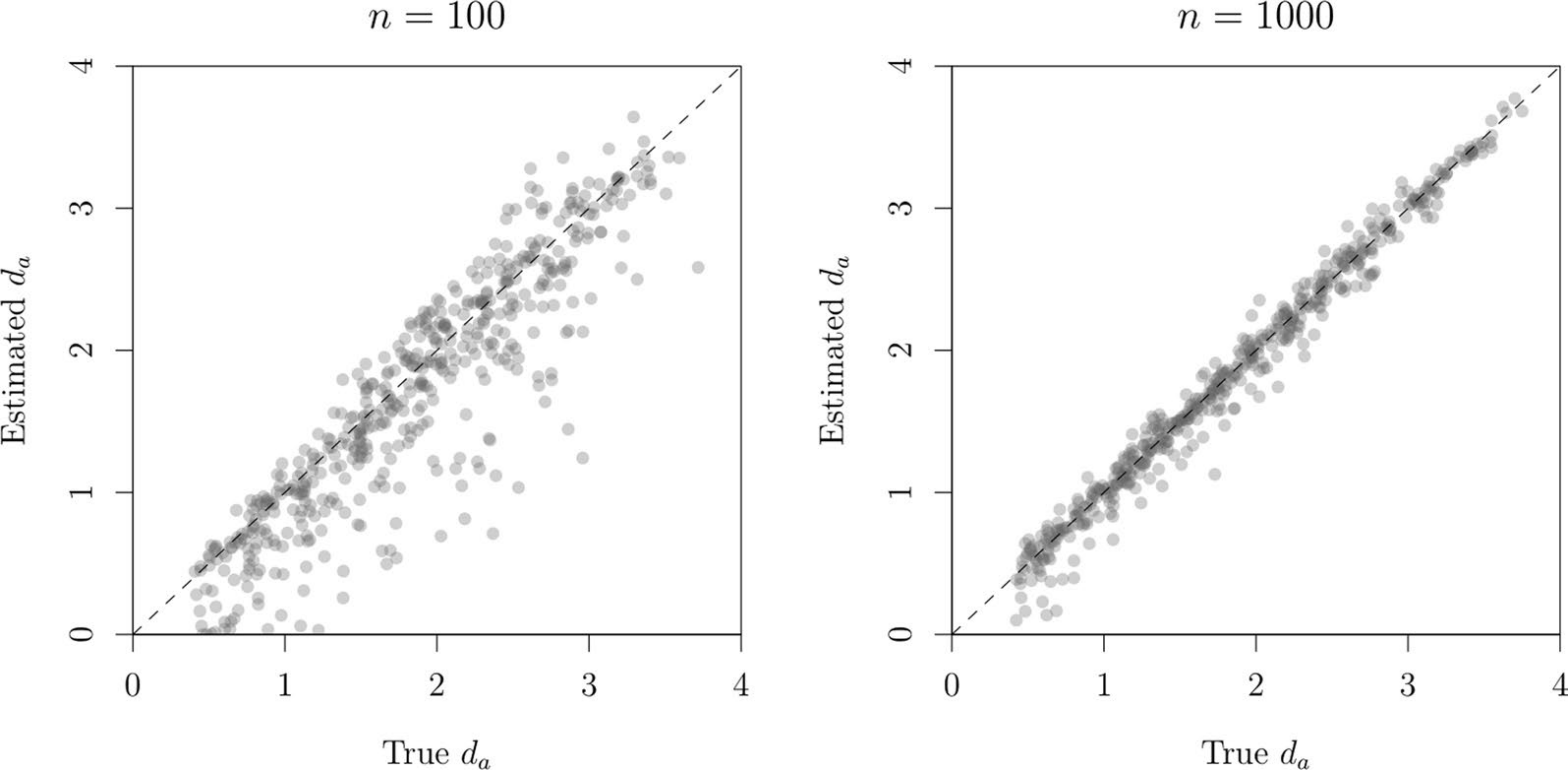
True and estimated $d_a$ from the SDT$_{\tau 4, \emptyset }$ model, under different *n* per lineup condition

Our first simulation investigated the ability to estimate discriminability—as quantified by $d_a$—from binary judgments: We simulated binary response data from SDT$_{\tau 4, \emptyset }$ model, in which we randomly sampled $\mu _T$ and $\sigma ^2_T$ values from independent uniform distributions with ranges [0.2, 1.40] and [0.3, 1.2], respectively. The remaining parameter values were the ones estimated from confidence ratings (see the bottom half of Table 3). Two-hundred simulated datasets (each its own $\mu _T$ and $\sigma ^2_T$ values) were generated, with 100/1000 responses per lineup condition. As illustrated in Fig. 11, the true data-generating $d_a$ are closely followed by their estimated counterparts, with rank correlations of 0.91 and 0.99 for $n = 100$ and 1000, respectively.

In a second simulation, we evaluated the ability to correctly identify the causes behind the differences between two groups: Are they caused by differences in discriminability, response bias, or both? In order to answer this question, we fit different versions of the SDT$_{\tau 4, \emptyset }$ model to binary response data coming from two groups A and B. The different model versions implemented four different hypotheses:

1. Groups A and B do not differ (None),
2. Groups A and B differ in terms of discriminability (Discriminability),
3. Groups A and B differ in terms of response criteria (Criteria),
4. Groups A and B differ in terms of both discriminability and response criteria (Both).

Binary response data were generated from these different models, once again using the SDT$_{\tau 4, \emptyset }$ parameters estimated from confidence ratings as a basis (see the bottom half of Table 3). Scenarios in which group B has lower discriminability were introduced by decreasing $\mu _T$ by 10% (i.e., a difference of approximately 0.08), whereas differences in response criteria were obtained by shifting $\tau _0$ by $\pm 0.20$ across all sequence positions. Finally, we assumed 500/1000 responses per lineup condition in each group (for each group, there are six target-present lineup conditions and one target-absent lineup condition; see Table 1). These differences, as well as the sample sizes, strike us as reasonable reference values when taking consideration previous reports (e.g., Dunn et al., 2022; Wilson et al., 2019). Two-hundred datasets were generated per scenario. Table 4 reports the frequencies with which the null hypotheses that discriminability and response criteria are equal across groups were found to be statistically significant ($p < .05$).^16^ These frequencies track the data-generating models quite well. Importantly, note that shifts in response criteria are rarely being misinterpreted as differences in discriminability (for a contrasting state of affairs in recognition memory, see Rotello et al., 2008; Verde et al., 2006).

In a third and final simulation, we evaluated the ability to detect differences in $\mu _T$ across sequence positions, as postulated by the DFD hypothesis. Essentially, we replicated the simulation reported in the previous section (see Fig. 10), only that this time we are fitting binary responses instead of confidence judgments. For data-generating parameters $\alpha _{2-6} = 1.10$ and 1.50 (i.e., after the first sequence position, discriminability increases by 10% and 50%, respectively), the percentages of statistically significant $\Delta G^2$ values comparing SDT$_{\tau 4,\,\emptyset }$ and SDT$_{\tau 4,\,\mu 1}$ models were $10\%$, and $30\%$. For reference, the frequency of statistically significant results for $\alpha _{2-6} = 1.10$, when fitting confidence ratings, was 97% (see the right panel of Fig. 10).

**Table 4:** Hypothesis testing results from simulated binary judgments

True difference	Equality hypothesis rejected ($p < .05$)			
	*n* per lineup condition = 500		*n* per lineup condition = 1000	
	Discriminability (%)	Criteria (%)	Discriminability (%)	Criteria (%)
None	5	4	6	5
Discriminability	**92**	4	**99**	7
Criteria (−)	6	**79**	5	**99**
Criteria ($+$)	7	**71**	7	**96**
Both (−)	**86**	**84**	**99**	**99**
Both ($+$)	**78**	**74**	**99**	**98**

Two-hundred datasets were simulated per scenario. The percentages in bold denote the correct rejections. Symbols $+$ and $-$ in parentheses indicate whether the criteria in group B are more lenient or stricter, respectively

Altogether, these simulation results show that binary judgments coming from sequential lineups can be used to obtain valid SDT characterizations and conduct simple between-group comparisons. However, the use of binary judgments severely impairs our ability to detect differences in discriminability across sequence positions, as postulated by the DFD hypothesis. To be clear, none of these simulation results puts into question the overall value of confidence judgments in the study of eyewitness identifications—they only delineate the circumstances in which confidence ratings are not strictly necessary (see also Footnote 5).

### The merit of sequential lineups relative to showups

In their second experiment, Wilson et al. also collected responses from a *showup procedure*, in which participants are only shown a single face, which is either the target or a lure. The availability of such data allows us to directly compare the relative merits of the two procedures. We conducted a first comparison by directly fitting the showup data jointly with the data from the first sequence position.

**Fig. 12:**
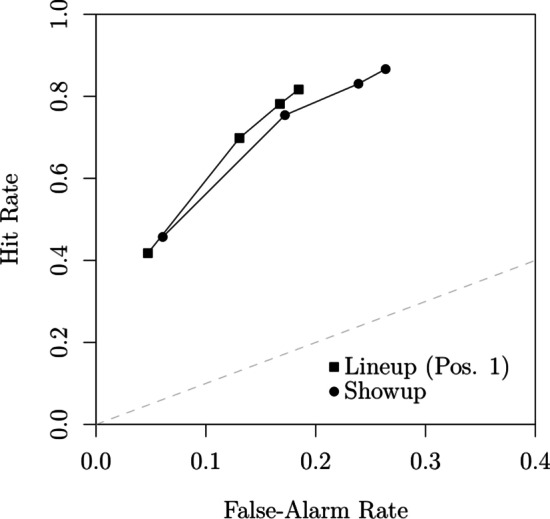
Confidence rating ROCs (from Wilson et al., 2019) obtained in position 1 of the sequential lineup procedure and the showup procedure

The SDT model used allowed $\mu _T$ and $\sigma ^2_T$ as well as the confidence criteria to differ across procedures. The badness of fit of this model was acceptable ($G_{\text {d}f = 4}^2 = 5.99$, $p = .20$). Restricting $\mu _T$ and $\sigma ^2_T$ to be the equal across conditions produced a negligible increase in misfit ($\Delta G_{\text {d}f = 2}^2 = 2.29$, $p = .32$), whereas restricting $\tau _0$ yielded a large increase ($\Delta G_{\text {d}f = 1}^2 = 29.31$, $p < .0001$). These results are corroborated by the ROC functions associated with the two conditions, which are illustrated in Fig. 12. The fact that discriminability was found to be the same across procedures constitutes an argument against the use of sequential procedures, at least when considering showups as an alternative. As illustrated on the right panel of Fig. 3, the ROCs associated with later sequence positions are not only dominated by their antecedents, their maximum hit rate steadily decreases. Assuming that each target position is equiprobable, it follows that the expected/average ROC function for sequential lineups will be dominated by the ROC function for showups.

However, a different and arguably more nuanced picture is obtained when taking into consideration the different possible outcomes associated with each procedure, their respective utilities, and the prior probability of suspect being guilty (Ceci & Friedman, 2000; Clark, 2012). Table 5 reports the probabilities of the different response outcomes for both sequential lineups and showups (these outcomes were described earlier in the Introduction). Based on all these values, we can compute the *expected utility* of each procedure:

$$\begin{aligned} U_{\rm procedure}&= P_{\rm guilty}\sum _{i=1}^3 P_{TPi} \times U_{TPi} + (1-P_{\rm guilty})\sum _{i=1}^3 P_{TAi} \times U_{TAi} \end{aligned}$$

**Fig. 13:**
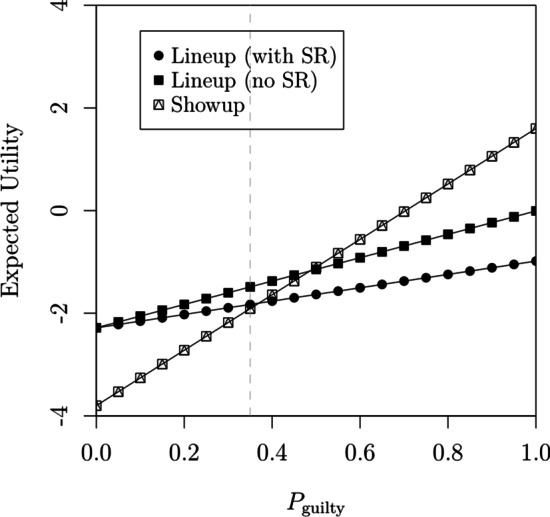
Expected utilities associated with different procedures, as a function of the probability that the suspect is guilty ($P_{\rm guilty}$) and the outcome frequencies reported in Table 5. SR = stopping rule. The gray dashed line corresponds to the $P_{\rm guilty}$ estimate reported by Wixted et al. (2016)

Using the utilities specified in Table 5 while varying the probability of a suspect being guilty ($P_{\rm guilty}$), we can compare the merit of sequential lineups relative to showups. As shown in Fig. 13, *the expected utility of sequential lineups is greater when the probability of the suspect being guilty is moderate or small (less than .40)*. A second look at Table 5 helps us to see why: The recognition of innocent suspects is lower for sequential lineups than showups (11.6% vs. 26.4%) due to the protective effect of known lures. However, this advantage can be cancelled or even overtaken by the considerable amount of known lures that are incorrectly identified in target-present and target-absent lineups (38.8% and 57.9%).

*But what if we remove the stopping rule?* In order to investigate this alternative scenario, we considered the *last* “yes” response that participants made instead of the first one. Note that this change can only affect outcome frequencies in target-present lineups. In the case of target-present lineups, removing the stopping rule led to a considerable increase in the hit rates ($+19.9\%$) and an equivalent decrease in false alarms. This difference shows that *removing the stopping rule is generally beneficial, allowing individuals to correct previous mistakes*. This difference leads to noticeable improvements in terms of expected utility (see Fig. 13).

Based on these comparisons, we do not think that there is a clear case against sequential lineups relative to showups, especially if we consider the possibility of removing the stopping rule. Whether or not sequential lineups should be preferred to showups depends on numerous factors, such as the probability that the suspect is guilty, which can vary dramatically from jurisdiction-to-jurisdiction, situation-to-situation, and detective-to-detective (cf. Lampinen et al., 2019). For reference, Wixted et al. (2016) reported an estimated probability of .35, which, if taken at face value, renders sequential lineups equiparable or superior to showups, depending on whether or not the stopping rule is imposed (see Fig. 13).

**Table 5:** Outcome percentages and utilities for the sequential lineup and showup conditions (data from Wilson et al., 2019)

Outcome	Utility	% Lineups (with stopping rule)	% Lineups (no stopping rule)	% Showups
*TP1* (Hit)	2	52.3	66.2	86.6
*TP2* (FA, Known Lure)	− 5	38.8	24.9	–
*TP3* (Miss)	− 1	8.9	8.9	13.4
*TA1* (FA, Known Lure)	− 1	57.9		–
*TA2* (FA, Innocent Suspect)	− 20	11.6		26.4
*TA3* (Miss)	2	30.5		73.6

FA, false alarm. Please note that outcomes *TP2* and *TA1* are not possible in the case of showups

### Discussion

The reanalysis of Wilson et al. (2019) allowed us to tackle a number of foundational issues regarding the application of SDT to sequential lineups. First, we showed that SDT can provide a satisfactory characterization of people’s eyewitness judgments without obliterating the sequential structure of the data or call for a large number of free parameters (Q1). These results contrast with previous attempts in which the models considered yielded considerable misfits (e.g., Kaesler et al., 2020; Wilson et al., 2019). We were also able to establish the relative merits of binary and confidence rating judgments with regards to estimating discriminability and testing hypotheses: Although incorporating confidence ratings is generally advantageous, there are a number of circumstances in which binary judgments are enough to obtain a valid SDT characterization (Q2).

Our reanalysis of Wilson et al.’s (2019) data suggests that people’s response criteria change across the lineup sequence and that they are affected by the rejection of the target. These results are consistent with the learning process postulated by Turner et al. (2011): When the target is rejected, this leads the witness to change their belief regarding the latent strength of lures. Namely, the witness will believe that lures are generally “more familiar” than they actually are. This change can produce a conservative shift in the response criterion, especially if said criterion is trying to maintain a certain degree of response bias (Kantner & Lindsay, 2012; 2014; see also Fig. 2). It is worth pointing out that this observed pattern is at odds with previous experimental reports coming from the recognition memory literature (Malmberg and Annis, 2012).

In contrast, we found minimal, nonsignificant effects of sequence position at the level of discriminability. These results are strengthened by complementary simulations showing that the observed differences in model performance (when fitting confidence ratings) are consistent with the null hypothesis and inconsistent with the presence of an effect, even if small (see Fig. 10). In short, the testing conditions—in terms of both experimental design and the models implemented—are well suited for detecting the kind of differences in discriminability postulated by the DFD hypothesis (Q3). But although these results speak against the DFD hypothesis, it would be a mistake to generalize beyond this specific procedure or even this specific study. It is clear from earlier discussions comparing simultaneous and sequential lineups that the learning opportunities postulated by the DFD hypothesis were already expected to be limited in the latter case (see Wilson et al., 2019; Wixted & Mickes, 2014). There is also the fact that Wilson et al.’s study did not actually impose a stopping rule; its imposition was emulated afterwards when structuring the data for analysis. The generalizability of these results will be put to the test in the section below, where we reanalyze the data from a follow-up study by Dunn et al. (2022) in which a stopping rule was enforced. Interestingly, we observe discrepancies between the two studies that need to be addressed. Beyond generalizability, we will tackle a number of issues also raised by Dunn et al., regarding the use of aggregate data and its inferential risks.

Finally, we compared sequential lineups with showups. The discriminability estimated in both procedures was essentially the same, which speaks against the use of sequential lineups altogether given the effects of target sequence positioning (see the left panel of Figs. 3 and 9). However, an alternative comparison based on expected utilities provides a more nuanced picture, indicating specific scenarios in which sequential lineups are preferred, especially if the stopping rule is removed (Q4). To be clear, our comparisons of expected utilities are based on *arbitrary* utility values assigned to each possible outcome. That being said, we believe that the present utility values are more sensible than simply setting the utilities of all un/desirable outcomes to $\pm 1$, as done in previous work (e.g., Smith, Lampinen, Wells, Smalarz, & Mackovichova, 2018). The utility values adopted here capture some of our most basic intuitions regarding the relative severity of the different outcomes: On the one hand, we perceive the incorrect identification of an innocent suspect as a tragic outcome whose avoidance should be prioritized. On the other hand, we find the incorrect identification of a known lure in a target-present outcome (which favors the guilty suspect) to be a worse outcome than failing to recognize anyone, or incorrectly recognizing a known lure in a target-*absent* lineup. Researchers are of course encouraged to consider alternative utilities, provided that some kind of justification is provided.

## Revisiting Dunn et al. (2022)

Dunn et al.’s (2022) experimental design differs from Wilson et al.’s (2019) in two major ways: First, they relied on an asymmetric ratio (approximately 4 to 1) of responses collected from target-present and target-absent lineups. Their rationale was that a biased ratio, which increases the total number of responses to target-present lineups, is preferable when attempting to characterize phenomena related to target positioning. Second, their study imposed a stopping rule, such that the sequential lineup procedure terminated as soon as a participant made their first “yes” response. The data were modeled exactly as in the case of Wilson et al., but in light of the results reported earlier, we focused our efforts entirely on the confidence rating data and on the SDT models with unconstrained response criteria.

**Table 6:** Model selection results and parameter estimates (Reanalysis of Dunn et al., 2022)

Confidence rating judgments									
Model		Parameters		$G^2$		AIC		BIC	
SDT$_{\tau 4,\,\emptyset }$		30		215		275		482	
SDT$_{\tau 4,\,\mu 1}$		31		171		**233**		**446**	
SDT$_{\tau 4,\,\sigma 1}$		31		171		**233**		**446**	
SDT$_{\tau 4,\,\mu 2}$		36		169		240		488	
SDT$_{\tau 4,\,\sigma 2}$		36		169		240		488	

SDT$_{\tau 4,\, \emptyset }$		SDT$_{\tau 4,\, \mu 1}$		SDT$_{\tau 4,\, \sigma 1}$		SDT$_{\tau 4,\, \mu 2}$		SDT$_{\tau 4,\, \sigma 2}$	
$\mu _T$	0.88	$\mu _{T}$	0.78	$\mu _{T}$	0.78	$\mu _{T}$	0.78	$\mu _{T}$	0.78
$\sigma ^2_T$	0.47	$\sigma ^2_{T}$	0.46	$\sigma ^2_{T}$	0.46	$\sigma ^2_{T}$	0.46	$\sigma ^2_{T}$	0.46
$\tau _{0,1}$	0.39	$\alpha _{1}$	1	$\xi _{1}$	1	$\alpha _{1}$	1	$\xi _{1}$	1
$\tau _{0,2}$	0.50	$\alpha _{2-6}$	1.17	$\xi _{2-6}$	0.73	$\alpha _{2}$	1.17	$\xi _{2}$	0.73
$\tau _{0,3}$	0.44	$\tau _{0,1}$	0.41	$\tau _{0,1}$	0.41	$\alpha _{3}$	1.16	$\xi _{3}$	0.74
$\tau _{0,4}$	0.43	$\tau _{0,2}$	0.50	$\tau _{0,2}$	0.43	$\alpha _{4}$	1.19	$\xi _{4}$	0.71
$\tau _{0,5}$	0.42	$\tau _{0,3}$	0.44	$\tau _{0,3}$	0.38	$\alpha _{5}$	1.17	$\xi _{5}$	0.73
$\tau _{0,6}$	0.42	$\tau _{0,4}$	0.43	$\tau _{0,4}$	0.37	$\alpha _{6}$	1.12	$\xi _{6}$	0.80
$\delta$	0.49	$\tau _{0,5}$	0.44	$\tau _{0,5}$	0.37	$\tau _{0,1}$	0.41	$\tau _{0,1}$	0.41
$\lambda$	0.13	$\tau _{0,6}$	0.43	$\tau _{0,6}$	0.37	$\tau _{0,2}$	0.50	$\tau _{0,2}$	0.43
$\omega$	0.84	$\delta$	0.45	$\delta$	0.39	$\tau _{0,3}$	0.44	$\tau _{0,3}$	0.38
$\eta$	-0.17	$\lambda$	0.16	$\lambda$	0.16	$\tau _{0,4}$	0.43	$\tau _{0,4}$	0.36
$\gamma$	0.01	$\omega$	0.83	$\omega$	0.83	$\tau _{0,5}$	0.43	$\tau _{0,5}$	0.37
		$\eta$	-0.17	$\eta$	-0.17	$\tau _{0,6}$	0.42	$\tau _{0,6}$	0.38
		$\gamma$	0.01	$\gamma$	0.01	$\delta$	0.44	$\delta$	0.38
						$\lambda$	0.14	$\lambda$	0.13
						$\omega$	0.84	$\omega$	0.84
						$\eta$	-0.17	$\eta$	-0.17
						$\gamma$	0.02	$\gamma$	0.02

The best-performing values according to AIC and BIC are underlined and in bold font

### Analysis of confidence judgments

**Fig. 14:**
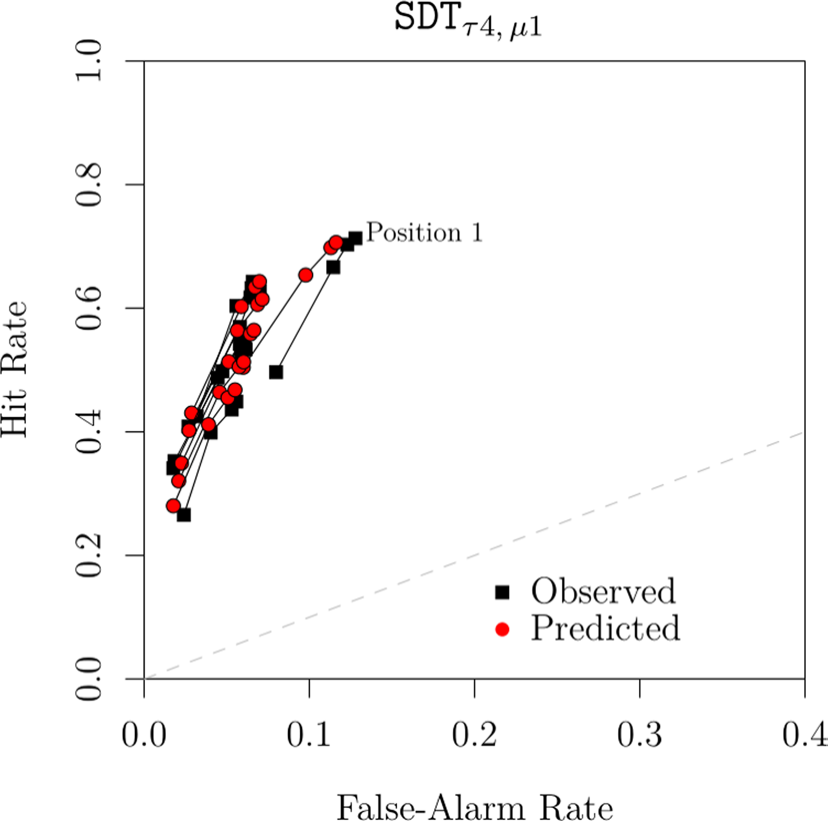
Observed confidence rating ROCs and the corresponding predictions from SDT$_{\tau 4, \mu 1}$ (Reanalysis of Dunn et al., 2022)

The modeling results reported in Table 6 show that, contrary to what we found with Wilson et al.’s (2019) study, both models SDT$_{\tau 4,\,\mu 1}$ and SDT$_{\tau 4,\,\sigma 1}$ outperform their competitors. These two models were found to provide a competent characterization of the data ($G_{\text {df} = 137}^2 = 171$, $p = .03$), especially when taking into consideration the large sample size. For reference, the observed ROCs and the predictions made by model SDT$_{\tau 4,\,\mu 1}$ are contrasted in Fig. 14. For both SDT$_{\tau 4,\,\mu 1}$ and SDT$_{\tau 4,\,\sigma 1}$, restricting $\sigma ^2_T = \sigma ^2_L = 1$ led to large increases in misfit ($\Delta G_{\text {df} = 1}^2 = 89.19$, $p < .0001$). This result corroborates earlier findings that $\sigma ^2_T < \sigma ^2_L$ in the context of eyewitness judgments (e.g., Wixted et al., 2018).

An inspection of the parameters estimates of models SDT$_{\tau 4,\,\mu 1}$ and SDT$_{\tau 4,\,\sigma 1}$ in Table 6 shows that $\alpha _{2-6} = 1.17$ and $\beta _{2-6} = 0.73$. Both estimates indicate a clear increase in discriminability after the first sequence position. These effects should be seen as optimistic estimates though: A visual inspection of the ROCs in Fig. 14 shows that the predicted ROC for position 1 *dominates* its observed counterpart. What this means is that discriminability in sequence position 1 is being overestimated, which in turn suggests that the difference between this sequence position and the others is being inflated (but see Footnote 13). We also note that the learning effect is limited in scope: The improvements offered by models and SDT$_{\tau 4,\,\mu 2}$ SDT$_{\tau 4,\,\sigma 2}$ are negligible at best (largest $\Delta G_{\text {df} = 4}^2 = 2.32$, smallest $p = .68$) and the estimated changes in discriminability do not suggest any kind of monotonic pattern (see Table 6). If anything, discriminability appears to reach a plateau after an initial increase. We will return to this issue in the section below.

**Fig. 15:**
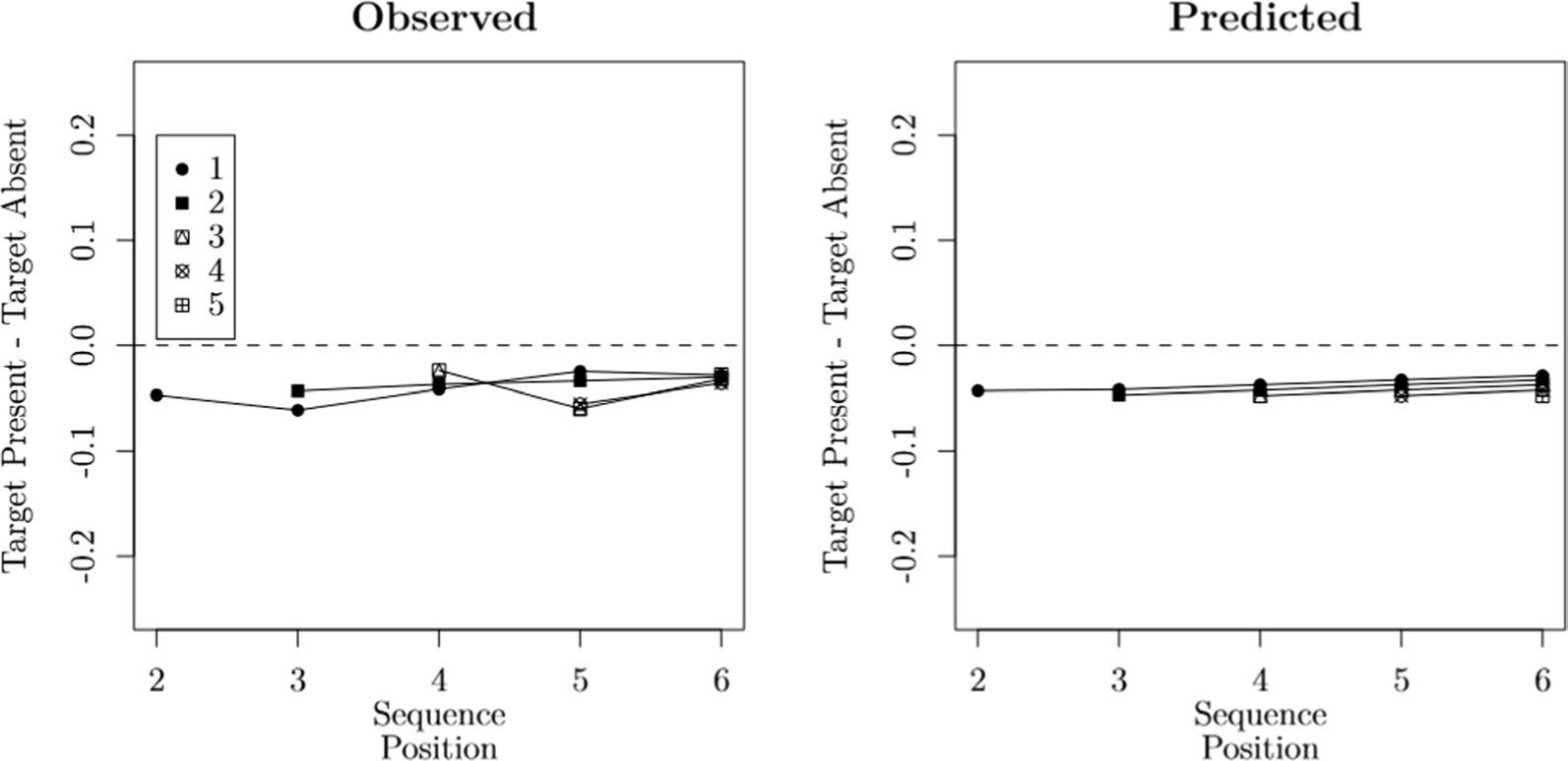
Differences in the conditional probability of responding “yes” in sequence position *i* between target-present and target-absent lineups. The different symbols distinguish the preceding position taken by the target in target-present lineups

Turning our attention to response criteria, we also observe some discrepancies relative to Wilson et al. (2019): Restricting the binary response criteria $\tau _0$ in the SDT$_{\tau 4,\,\mu 1}$ and SDT$_{\tau 4,\,\sigma 1}$ models to be fixed across sequence positions did *not* lead to statistically significant increases in badness of fit (largest $\Delta G_{\text {df} = 5}^2 = 8.41$, smallest $p = .13$). We found large differences in misfit when setting $\delta =0$ ($\Delta G_{\text {df} = 1}^2 = 62.86$, $p < .0001$) but not when setting $\lambda = 0$ ($\Delta G_{\text {df} = 1}^2 = 2.89$, $p = .09$). Figure 15 illustrates the differences in proportions of “yes” responses (observed and predicted) when conditioning on a prior target rejection. These differences, which are similar but smaller than the ones found in Wilson et al.’s data (see Fig. 7), show a stable decrease in “yes” responses after the target is rejected.

Altogether, the modeling results obtained with Dunn et al.’s (2022) data support the idea that people learn how to make recognition judgments throughout the lineup sequence. This learning is manifested in two different ways: On the one hand, we found an increase in discriminability, in line with the DFD hypothesis. On the other hand, we also saw changes in the response criteria when participants incorrectly rejected the target, which is consistent with the learning process proposed by Turner et al. (2011). But how can the aforementioned change in discriminability be reconciled with the “null effect” obtained with Wilson et al.’s (2019) data? Our attempts to provide an answer to this question are detailed in the section below.

### Contrasting and reconciling the two studies

One of the major differences between the Wilson et al.’s (2019) and Dunn et al.’s (2022) studies was the ratios of responses to target-present and target-absent lineups collected. At first glance, this difference appears to offer an explanation for the observed discrepancies at the level of discriminability: The biased ratio adopted by Dunn et al. is particularly advantageous when trying to characterize effects related to target positioning, which is the exact kind of effect being postulated by the DFD hypothesis—discriminability increasing across sequence positions. This explanation strikes us as implausible for two reasons: First, the two datasets have clear qualitative differences in terms of the ROC curve for sequence position 1 (compare Figs. 9 and 14). Second, the simulation results reported in the previous section show that even small differences—considerably smaller than the one found in Dunn et al.’s data—can be reliably detected with Wilson et al.’s experimental design.

A second experimental design difference that stands out is the way that each study handled participants’ “yes” judgments: Dunn et al.’s (2022) procedure imposed a stopping rule, whereas participants in Wilson et al.’s (2019) study were required to evaluate all the faces in the sequence. Recent work by Horry et al. (2021) has shown that the presence of a stopping rule induces more conservative response criteria. A comparison of the response criteria estimated from the two datasets replicates this finding, with Dunn et al.’s estimates being more conservative. When cast in terms of log-likelihood ratios (which take into account discriminability differences; see Macmillan & Creelman, 2005, Chap. 2), the criteria from Wilson et al. *were always more lenient* than their Dunn et al. counterparts, with an average difference of -0.67 (paired Wilcoxon test: $W=0$, $p=.03$).

Aside from response criteria, this procedural difference also invites different explanations for the discrepancies found at the level of discriminability: One possibility is that the absence of a stopping rule in Wilson et al.’s (2019) study discourages people from seriously engaging with the task. After all, it introduces the notion that mistakes can be corrected down the line. In principle, a reduced engagement could compromise learning throughout the lineup. Fortunately, there is one way to directly test this hypothesis: As previously discussed, the second experiment reported by Wilson et al. also collected responses from a showup procedure. Importantly, this procedure does not give any opportunity for corrections later on. If participants are expected to be less engaged in sequential lineups without a stopping rule, *then performance in the first sequence position should lead to a lower discriminability relative to the showup condition*. But as discussed earlier, the two conditions do not differ in terms of discriminability (see Fig. 12). This means that the data do not provide any support to the notion that the observed discrepancy between Wilson et al. and Dunn et al. is due to some kind of reduced engagement caused by the lack of a stopping rule.

However, we can think of a second possible explanation that puts the onus on Dunn et al.’s (2022) study: Perhaps, a small portion of their participants selected the first face that they encountered in order to complete the experiment as fast as possible. This explanation highlights one of the issues that arises from the use aggregate data. Namely, that *differences between participants might be mistaken for effects taking place at the individual level* (for a relevant discussion, see Regenwetter & Robinson, 2017). Dunn et al. raised a similar concern when discussing the effect of target rejections (which we will address later on). But there is no reason for circumscribing these concerns to that specific effect. The feasibility of this explanation in the present case was evaluated by fitting an extended version of the SDT$_{\tau 4,\,\emptyset }$ model. This extended model assumes that participants’ responses follow a *two-component mixture distribution*: With probability $\pi$, a sampled participant is “*serious*” such that their judgments are assumed to follow the SDT$_{\tau 4,\,\emptyset }$ model. With complementary probability $1-\pi$, a sampled participant is “*unserious*,” such that they *always* select the first face that they encounter in the sequence. Confidence rating probabilities for “unserious” participants were determined by the following state-mapping function, which is governed by a single parameter $\zeta > 0$. Let $k=1, \dots , K$ denote the *K* confidence levels associated with a “yes” response, with $k=1$ denote minimum confidence, and $k=K$ maximum confidence (for a similar approach, see Klauer & Kellen, 2010):

$$\begin{aligned} P("k" \mid {\texttt {"yes"}}, \text {unserious}) = \frac{\exp (-\zeta k)}{\sum _{k^\prime =1}^{K}\exp (-\zeta k^\prime )}. \end{aligned}$$

The ability of this extended model to describe the observed data was found to be comparable to the SDT$_{\tau 4,\,\mu 1}$ model, with $G^2 = 167$ (AIC $= 231$ and BIC $= 452$). The two models make virtually identical predictions. Parameter estimates for the extended SDT$_{\tau 4,\,\emptyset }$ model were $\pi = .95$ and $\zeta = 0.89$, which indicate that the observed increase in discriminability (which is likely to be an overestimate, as discussed earlier) can be attributed to 5% of the participant sample invariably choosing the first face that they encountered with relatively low confidence (for $k=1, 2, 3, 4$, the confidence rating probabilities were .61, .25, .10, and .04, respectively).

In response, one could argue that it is not very surprising that a change in discriminability can be (re)described in terms of a change in mixture weights (see DeCarlo, 2010; Province & Rouder, 2012). The point is well taken, but it overlooks the circumstances behind this alternative characterization: The enforcement of a stopping rule in Dunn et al.’s (2022) sequential lineup procedure—and the lack thereof in Wilson et al.’s (2019) procedure—is what motivated this explanation in the first place. Second, this alternative explanation represents one of the many kinds of aggregation biases that could be present, biases that also concerned Dunn et al. (2022). We see no a priori reason to treat the present case as less plausible or legitimate than any other kind of aggregation bias, such as the possibility of “selection biases” on the effect of target rejections. Third, the characterization offered by the SDT$_{\tau 4,\,\mu 2}$ model for Dunn et al.’s data suggests a 17% increase in discriminability immediately after the first face is rejected *but no clear changes afterwards*—discriminability basically reaches a plateau (see Table 6; see also Dunn et al., Figure 3a). What this means is that, if we are in fact dealing with an active learning process, then the conditions in which it manifests itself turn out to be dramatically narrow. Fourth, there is the fact that this learning process is somehow absent in Wilson et al.’s data, even though there is no reason to expect its absence there, nor should there be any difficulty in detecting its presence. Finally, this reaction overlooks a very clear message: *that only five percent of the online participant sample are enough to distort the data in the direction of the DFD hypothesis*. In order to outright dismiss this alternative explanation, researchers would have to possess a degree of experimental knowledge/control that is arguably unrealistic.

### Dissolving the between-/within-subjects distinction in sequential lineup modeling

The previous section reiterated the inferential risks associated with relying on aggregate data. Some of these risks were discussed by Dunn et al. (2022), who tried to remedy them by conducting a so-called within-subjects analysis of Wilson et al.’s (2019) data. This analysis, which forfeited any kind of stopping rule emulation and modeled the *entire sequence of six responses* produced by each participant, was contrasted with the “between-subjects” analysis that enforced it. Note that according to Dunn et al.’s classification, all of the model comparisons reported in the present manuscript up to this point are “between-subjects” analyses. The results obtained in their “within-subjects” SDT model analysis corroborated some of the results obtained with its “between-subjects” counterparts, such as the effect of rejecting the target on response criteria (see Fig. 7). But whereas their “between-subjects” analysis found no effect of sequence position on discriminability (consistent with the present results), their “within-subjects” analysis found* discriminability to decrease* as the lineup sequence unfolded. Dunn et al. attributed this discrepancy to the presence of output interference or item noise effects (Criss et al., 2011; Osth et al., 2018).

We disagree with the understanding behind this between-/within-subjects distinction. As we will show below, it is a mistake to think that this “within-subjects” analysis offers some kind of alternative perspective: When properly construed, it becomes clear that one analysis is a *proper subset* of the other. This relationship appears to be overlooked because of additional invariance assumptions being enforced at the level of the data (i.e., enforced by the aggregation steps conducted). It turns out that these assumed invariances are what ultimately drives the discrepancies between both analyses. These issues are discussed in detail below, as they demonstrate how careful researchers must be when dealing with aggregate lineup data.

Let us begin with the between/within-subjects distinction. This distinction traditionally refers to the comparison between different groups of individuals viz-à-viz the comparison of the same group of individuals across experimental conditions. But this contrast cannot be applied here, given that the “between-subjects” analysis also considers the responses made by the same participant across different conditions (sequence position, target vs. lure). What distinguishes both analyses is the *censoring* imposed by the stopping rule: Whereas one analysis only includes the first “yes” response, the other includes the entire sequence of responses. Consider the following censored response sequence, in which the item in sequence position 4 is (the first to be) recognized and the uncensored sequences of length six that can stem from it:
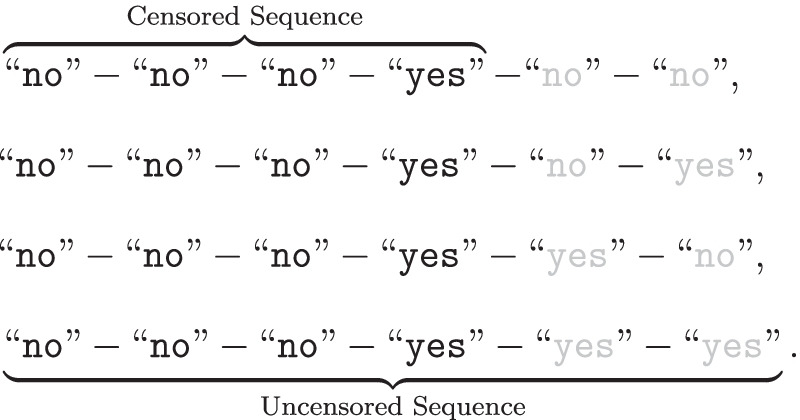


Going from censored to uncensored sequences increases the total number of unique sequences that can be observed in a given lineup. For lineups of length six, we have to consider how each of the seven censored patterns can be expanded, which ends up amounting to $1 + \sum _{i=1}^6 2^{i-1} = 64$ unique uncensored sequences.

The same SDT model framework described in Eqs. 1 and 2 can be used to characterize censored and uncensored response sequences. The major difference between both applications being that a number of multiplicative terms would be omitted in the former case, namely the terms denoting the probabilities of responses made after the first “yes.” However, it is would be unwise to assume that no further modifications are necessary. After all, we are dealing with an experimental task in which it is clear to everyone (participants included) that there is *at most one target* in a given lineup. We are not dealing with a randomized test list comprised of multiple studied and non-studied words, of the kind typically found in recognition memory studies. For instance, it is sensible to assume that a second “yes” response is only produced when the latent strength of a given face is greater than the previously recognized one, which implies a conservative shift of the response criterion. The possibility of discriminability being affected by a previous “yes” response is also plausible a priori, as evidenced by the numerous studies investigating the effects of repeating sequences and/or providing feedback (e.g., Godfrey & Clark, 2010; Horry et al., 2015; Palmer et al., 2010; Steblay et al., 2011a). In fact, rejecting this possibility a priori would result in a somewhat bizarre scenario in which we are willing to entertain the impact of rejecting the target on response criteria while simultaneously denying the possibility that a positive ID could in any way affect any of the eyewitness judgments made afterwards.

A simple way to incorporate the considerations above into our SDT models consists of allowing SDT parameters to change after a “yes” response has been made. Let $\{ {\varvec{\mu /\sigma }, \varvec{\tau _0}}\}$ denote the set of parameters operating up to the point a “yes” response is first made, and $\{ {\varvec{\mu ^\bigstar /\sigma ^\bigstar }}, \varvec{\tau ^\bigstar _0}\}$ denote the set of parameters operating afterwards. Now, let us consider the seven uncensored response sequences below, where “  ” denotes unspecified yes/no responses:
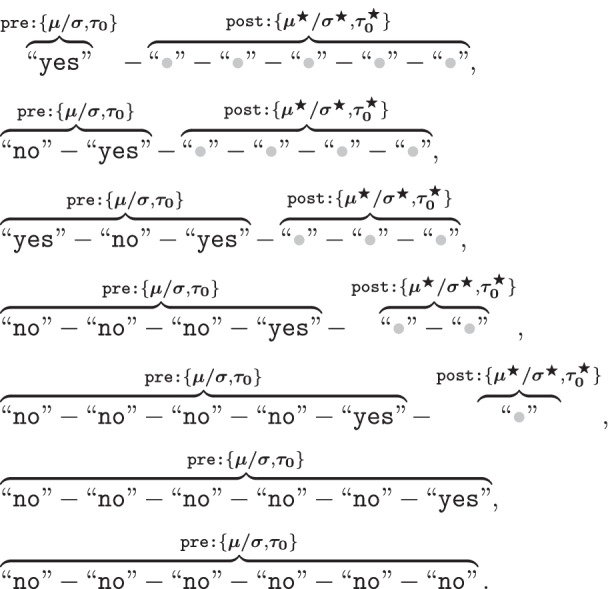
As previously mentioned, the sixty-four possible uncensored sequences are obtained when expanding the seven possible censored sequences beyond the first “yes” response. The seven sequences listed immediately above capture how this expansion takes place and how any uncensored sequence can be cast as the conjunction of two segments representing *pre*- and *post*-first-“yes” responses—${\texttt {pre}} \cap {\texttt {post}}$. Now, note that the probability of any given uncensored sequence can be expressed as $P({\texttt {pre}} \cap {\texttt {post}}) = P({\texttt {pre}}) \times P({\texttt {post}} \mid {\texttt {pre}})$. The first product term, which corresponds to the probability of the *censored* sequence segment, is governed by its own set of parameters, namely $\{\varvec{\mu /\sigma },\varvec{\tau _0}\}$. The second product term, a conditional probability, refers to responses made *after* the first “yes” response; these responses are governed by their own parameter set $\{\varvec{\mu ^\bigstar /\sigma ^\bigstar },\varvec{\tau ^\bigstar _0}\}$. This decomposition makes it clear why an analysis of uncensored sequences cannot be used as a form of corroboration—basically the same set $\{\varvec{\mu /\sigma },\varvec{\tau _0}\}$ of functionally independent parameters are being estimated in both analyses. In fact, any discrepancy between the estimates obtained with censored and uncensored sequences will be caused by the model’s inability to accurately describe $P({\texttt {post}} \mid {\texttt {pre}})$, in all likelihood due to constraints being imposed over $\{\varvec{\mu ^\bigstar /\sigma ^\bigstar },\varvec{\tau ^\bigstar _0}\}$.

The relationship between the two kinds of analyses is not obvious in Dunn et al.’s (2022) analyses because they did not fit their models to uncensored response sequences directly. Instead, they opted to focus on the (marginal) response probabilities associated with each sequence position. This approach throws away a considerable amount of sequential information, enforcing the assumption that, aside from past encounters with the target, participants’ responses at a given position are *independent and identically distributed* (i.i.d.).^17^ What this means is that very different sequences *are treated as equivalent* when determining $P("{\texttt {yes}}")$ for a given position. Sequences that, as discussed above, are likely governed by different sets of SDT parameters. For example, for sequence position 3, it treats the four sequences below as equivalent (the first being the only one considered when analyzing censored sequences):
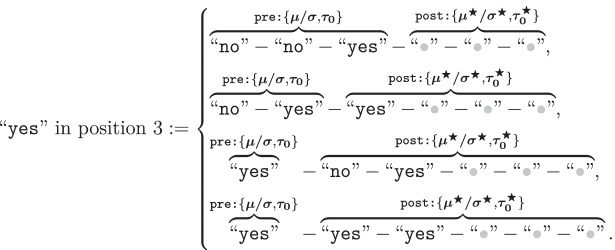


This i.i.d. assumption enforces a bizarre scenario—discussed earlier—in which any possible effect of prior responses is excluded a priori. Importantly, because this assumption is introduced in the way responses are represented in the data (marginal responses rather than sequences) the researcher is effectively unable to determine its violation by inspecting SDT model misfits (for extensive discussions, see Birnbaum, 2011, 2013). In order to evaluate the merits of this assumption, one needs to “unpack” the data and directly test the null hypothesis that the probability of a “yes” response on a given position *i* is the same across all of the possible preceding sequences (for a discussion, see Smith & Batchelder, 2008). It turns out that this hypothesis is at odds with the data. For example, in the case of “yes”/“no” responses to a target on position 3 ($G^2_{\text {df} = 3} = 72.17$, $p < .0001$). This result follows from the fact that the probabilities of “yes” response across the four possible sequences listed above are .84, .56, .50, and .64, respectively. In words, the probability of a “yes” response to a target at position 3 is considerably lower when participants gave at least one “yes” response before. A statistically significant violation of i.i.d is also obtained when looking at “yes” responses in position 3 of target-absent lineups, with proportions .19, .10, .13, and .22 ($G^2_{\text {df} = 3} = 72.17$, $p < .0001$).

**Fig. 16:**
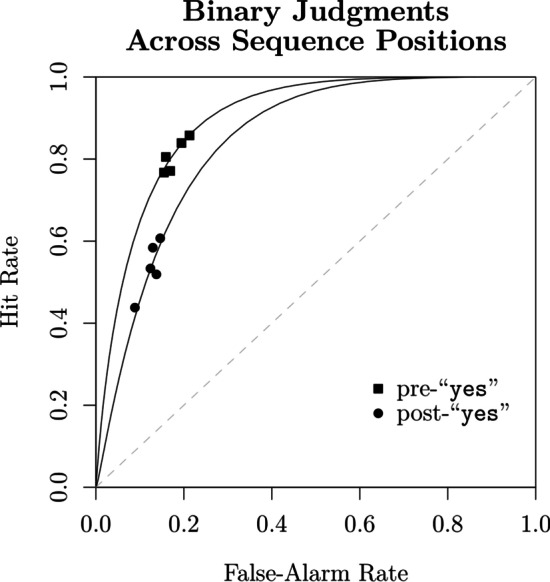
Binary response ROCs (from Wilson et al., 2019) obtained with marginal responses per sequence position (each point corresponds to a sequence position, from 2 to 6). Black squares denote cases in which no “yes” responses were made in any previous positions (pre-“yes”). Black circles denote the complementary cases in at least one “yes” response was previously made (post-“yes”)

These violations of the i.i.d. assumption help us understand the reason why Dunn et al. (2022) found *discriminability to decrease across sequence positions* in their “within-subjects” analysis, which they attributed to output interference or item noise (Criss et al., 2011; Osth et al., 2018). Based on the response proportions above, it appears that discriminability might be lower after a “yes” response has been made. In response, we conducted a more rigorous analysis that paired choice proportions for targets/lures in target-present/absent lineups at each sequence position, from 2 to 6. We unpacked each of these pairs in terms of whether or not a “yes” response was previously made (hence the exclusion of the first sequence position).^18^ These pairs can be cast as hit/false alarm pairs and fit with a traditional SDT model for binary choices (e.g., Bröder & Schütz, 2009; Dube & Rotello, 2012). Discriminability and response criteria were allowed differ if a “yes” response was previously made. Criteria were also allowed to differ as a function of sequence position. This model fit the data well ($G^2_{\text {df} = 2} = 6.78$, $p = .34$), as shown in Fig. 16. In terms of parameter estimates, the prior occurrence of “yes” responses was associated with *lower discriminability* ($d_a$ difference = 0.35; $\Delta G^2_{\text {df} = 2} = 12.12$, $p = .002$) as well as *stricter response criteria* (mean $\tau _0$ difference = 0.39; $\Delta G^2_{\text {df} = 5} = 30.11$, $p < .0001$).^19^

With these results as a backdrop, we now turn to the prevalence of previous “yes” responses across sequence positions, which can be extracted from Table 1: In the case of target-present lineups, the percentages were 20%, 33%, 45%, 57%, and 60%, for positions 2-6. In target-absent lineups, they were 18%, 31%, 45%, 57%, and 64%. What these percentages are telling us is that the more faces one has evaluated, the greater the chances that some of them were recognized along the way. Jointly, the model fits and the percentages provide a clear explanation of Dunn et al.’s findings: Scenarios in which a “yes” response was previously made are associated with lower discriminability; scenarios that become increasingly prevalent in later sequence positions. Collapsing these scenarios with those in which no “yes” response has been made before results in aggregate data that spuriously suggest a decrease in discriminability across sequence positions.

### Can target rejection effects be attributed to aggregation?

The invalidity of Dunn et al.’s (2022) “within-subjects” analysis does not dismiss the concerns that it tried to address. Among them was the possibility that the observed effects of target rejection on subsequent lineup positions might be due to “*selection biases*.” Their argument can be summarized as follows:

- Participants adopt different response criteria, which they hold throughout the lineup condition.
- The more conservative the criteria, the fewer identifications will be made overall.
- Therefore, it should be expected that the more conservative eyewitnesses will be overrepresented among those whose lineup has not yet been terminated by a target acceptance (i.e., more conservative eyewitnesses will be overrepresented in sequences where the target appears later on).

Dunn et al.’s (2022) concern can be assessed indirectly, by using simulations to evaluate the plausibility of the selection bias hypothesis: When considering well-defined synthetic scenarios, do we find the aforementioned selection biases producing the kinds of effects observed in Figs. 7 and 15? Our evaluation consisted of three different simulations, all using the SDT$_{\tau 1,\,\emptyset }$ model as a basis (according to this model, the discriminability and criteria of each individual are fixed throughout the lineup). For simplicity, we also assumed that $\sigma ^2_T =\sigma ^2_L = 1$:

- *Simulation 1:* We only allowed the response criterion $\tau _0$ to vary across eyewitnesses while fixing $\mu _T$ to 0.75. Each individual $\tau _0$ was sampled from a uniform distribution ranging from $-0.25$ and 1.
- *Simulation 2:* Both $\tau _0$ and $\mu _T$ varied across eyewitnesses. Each individual $\mu _T$ was sampled from a uniform distribution ranging from 0.10 to 1.40 (note that $\mu _T = -\mu _L$). Individual $\tau _0$ were sampled as described in *Simulation 1*.
- *Simulation 3:* Both $\tau _0$ and $\mu _T$ varied across eyewitnesses, but we also assumed that $\tau _0$ was a function of a *likelihood ratio* (*LR*; for a review, see Glanzer et al., 2009). Individual *LR*s were sampled from an uniform distribution ranging from 0.75 and 2, whereas $\mu _T$ values were sampled as described in *Simulation 2*.

**Fig. 17:**
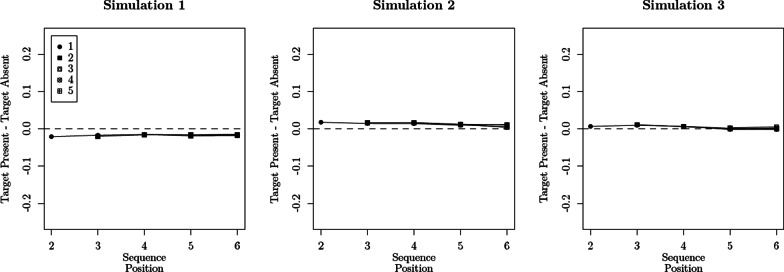
Simulated differences in the conditional probability of responding “yes” in sequence position *i* between target-present and target-absent lineups. The different symbols distinguish the preceding position taken by the target in target-present lineups

In each simulation, a total of 100,000 synthetic eyewitnesses were generated. Figure  17 illustrates the results in the exact same way as Figs. 7 and 15 did for Wilson et al.’s (2009) and Dunn et al.’s (2022) data. The only simulation that shows differences in line with the selection bias hypothesis is Simulation 1. However, the observed differences are considerably smaller, especially when compared against the results from Wilson et al. Both simulations 2 and 3, which can be seen as somewhat more realistic due to their introduction of individual differences at the level of discriminability, show an effect in the *opposite direction*. Both results show that individual differences in terms of discriminability and response criteria can interact in ways that ultimately render the selection bias hypothesis an implausible candidate explanation. The three simulations, together with the fact that we have a principled account for the effect of target rejection based on belief updating (see Fig. 2), lead us to believe that the effect of target rejections cannot be easily dismissed as the mere outcome of some kind of aggregation bias, that it is a real phenomenon taking place at the individual level.

### Discussion

Dunn et al.’s (2022) study makes two important contributions: First, it reports new experimental data that replicate Wilson et al. (2019) while imposing an actual stopping rule. The modeling results obtained with their data support an increase in discriminability, as postulated by the DFD hypothesis, as well as an effect of target rejection on response criteria, as expected by the learning process proposed by Turner et al. (2011). However, one might interpret the observed stability of response criteria across sequence positions as an indictment against this learning process. In our view, this would be a mistake, as one should keep in mind that different trends at the individual level (e.g., increasingly strict/liberal criteria) can cancel out at the aggregate level. What is common across all these heterogeneous individual cases is the expectation that the rejection of the target will result in an overestimation of the lure distribution, which in turn will induce the kind of conservative shifts that were found in both datasets.

A second important contribution is their explicit concern with the problems that aggregate data can pose. Motivated by similar concerns, we conducted additional analysis investigating how results could be distorted by the aggregation of heterogeneous individuals. One of our results showed that the observed increase in discriminability found in their data can be attributed to an unaccounted mixture of individuals (Q5). Further empirical work is necessary in order to determine the merits of this alternative explanation. In terms of response criteria, we did not find any reason to dismiss the effects of target rejection as the outcome of a “selection bias” that overrepresents conservative individuals (Q5). Finally, we critically evaluated the “within-subjects” analysis proposed by Dunn et al., which attempts to sidestep aggregation biases by removing the stopping rule from the sequential lineup procedure. Our critique lays out the reasons why this analysis should be dismissed in toto (Q6): It enforces i.i.d. assumptions that are not only unreasonable a priori but also rejected by the data when directly tested. Relaxing these i.i.d. assumptions does not provide any kind of relief though; it only leads to a redundant analysis of uncensored response sequences. To be clear, we are *not* arguing that there is no value in removing the stopping rule from sequential lineups—our expected utility analyses already showed that there is (see Fig. 13). We are simply saying that its removal cannot serve the role bestowed to it by Dunn et al.

## General discussion

SDT is arguably one of the greatest success stories in modern psychological research, with its application to recognition memory being a particularly notable example (for recent overviews, see Kellen & Klauer, 2018; Kellen et al., 2021; Rotello, 2019; Wixted, 2020). In light of this success, nobody should be surprised to see its application being regularly extended to new domains. In the case of eyewitness research, the inception of SDT modeling and the different concepts surrounding it (e.g., ROC functions) led to a renewed discussion on the relative merits of alternative procedures such as lineups and showups (see Gronlund et al., 2015). One of its most positive impacts has been the vindication of the idea that the relationship between people’s responses and theoretical concepts such as mnemonic discriminability is not trivial (e.g., Rotello & Chen, 2016; Starns et al., in press).

However, one should not confuse the merits of SDT modeling with the notion that it is a risk-free enterprise. After all, an excessive reliance on specific models and data structures can always lead to myopic or biased understandings (see Kellen, 2019; Spektor Kellen & Hotaling, 2018). For instance, the fact that one can subject eyewitness judgments to the same kinds of treatments given to recognition memory data does not mean that the two are essentially the same. Simply put, researchers need to take the unique characteristics of eyewitness procedures seriously. Fortunately, this issue has not gone unnoticed, as demonstrated by the recent efforts toward model tailoring and refinement (e.g., Dunn et al., 2022; Kaesler et al., 2020; Wilson et al., 2019; Wixted et al., 2018). The present work takes additional steps in this direction, by providing a comprehensive discussion on how to model sequential lineup data without compromising its “natural structure” or imposing unnecessary constraints, and the problems that can arise when failing to do so. For instance, in the case of Wilson et al. (2019), we saw how the unnecessary restriction of response criteria across sequence positions resulted in spurious evidence for changes in discriminability. In the case of Dunn et al. (2022), we saw how the introduction of questionable i.i.d. assumptions in their “within-subjects” analysis led to discrepant results and a misunderstanding of how they relate to “between-subjects” analyses.

According to Wilson et al. (2019), sequential lineups are “not well understood theoretically” (p. 122). This statement is corroborated by the discrepancies found between the two reanalyzed datasets and the challenges faced when trying to make sense of them. Our understanding of these discrepancies is that they are likely due to unaccounted aspects of the experimental design, such as the potential effect of enforcing a stopping rule on participant engagement. This possibility, together with observed benefits of removing the stopping rule, should be seen as encouragement for future work exploring different sequential lineup procedures. Our inability to sometimes go beyond (data- and simulation-informed) speculations is largely due to the reliance on aggregate data, which can yield results that might represent only a minority of individuals (Regenwetter & Robinson, 2017; Regenwetter et al., in press). Other issues also play a role, such as the use of different stimulus sets across studies, which introduce distorting “item effects” (see Singmann & Kellen, 2019; Trippas et al., 2018). Fortunately, there are well-established hierarchical extension methods for SDT models that could in principle address these concerns, provided that one can go beyond what the present data have to offer (see DeCarlo, 2011; Freeman et al., 2010; Pratte & Rouder, 2011; Trippas et al., 2018). In any case, one should not let the observed discrepancies detract from the consistencies that we were also able to identify, such as the lower latent strength variance for targets relative to lures (i.e., $\sigma ^2_T < \sigma ^2_L$), and the impact of target rejections on response criteria, which is line with the learning process postulated by Turner et al. (2011).

When searching for ways to obtain more informative lineup data, researchers are strongly encouraged to capitalize on SDT’s impressive track record in linking different types of judgments (see Gepshtein et al., 2020; Kellen et al., 2021; Meyer-Grant & Klauer, 2021). For example, Kellen et al. (2012) relied on the close relationship between ranking and yes-no recognition judgments to estimate criterion noise under minimal assumptions (for other examples of joint modeling, see Rouder & Batchelder, 1998; Chechile, 2004, Jang et al., 2009). This example is worth highlighting given the surging interest in eliciting rankings from eyewitnesses (see Brewer et al., 2020; Carlson et al., 2019). In the case of eyewitness identification, the joint modeling of simultaneous and sequential lineups appears to be quite promising, especially with regards to the testing of the DFD hypothesis: In their analysis of simultaneous lineups, Wixted et al. (2018) formalized the DFD hypothesis in terms of an “ensemble” SDT model that makes strong assumptions about the way that the latent strengths of the different options are considered together. In principle, the joint modeling of sequential and simultaneous lineups could be used to model the DFD hypothesis under more relaxed assumptions and establish closer links between the different eyewitness identification procedures.

On the other hand, researchers should also be mindful that the granularity of model-based characterizations is bounded by the richness of the data, which in turn are dependent on a number of real-world constraints (especially in applied settings). For instance, one can easily think of applied scenarios in which confidence ratings are simply not available or are perhaps unreliable. Fortunately, SDT models can be successfully applied to binary responses in order to answer a number of relevant questions, as demonstrated here. This possibility is an important reminder that models can serve multiple roles: They can provide a rich medium for theoretical development and refinement, but also serve as measurement tools. These roles are beholden to different standards (see Kellen, 2019; Navarro, 2019). For the latter role, it is often sensible to rely on simplified models, provided that they satisfy specific measurement desiderata (e.g., van Ravenzwaaij et al., 2017).

## Data Availability

Model code is available at https://osf.io/zgdfp/.

## Footnotes

1 Some studies create target-absent lineups by replacing the suspect with a specific “designated innocent suspect” chosen to be a close match to the perpetrator, but we will ignore this nuance for now (i.e., the distinction between outcomes *TA1* and *TA2* is not made), for the sake of simplicity.

2 In this toy example, the density estimates were based on 25 samples each along with a rectangular kernel. These samples were taken from Gaussian distributions with parameters $\mu _T = 1$, $\mu _L = 0$, and $\sigma ^2_T = \sigma ^2_L = 1$. The bandwidth value used was 0.75 and the update weight given to the updating item was .40 (for details, see Turner et al., 2011).

3 Because these distributions represent *beliefs* about the latent strength distributions, the increase in overlap caused by the rejection of the target does not mean that discriminability is actually reduced. Only that individuals believe that their discriminability is lower than it actually is.

4 For example, the frequencies of “yes” judgments for lures in positions 3 and 5 in target-present lineups include the “yes” judgments to lures in sequences where the target was presented in positions 1, 2, 4, and 6.

5 Please note that even if confidence ratings are unnecessary from a parameter estimation standpoint, they are most certainly not uninteresting nor worthless. As recently reviewed by Wixted and Wells (2017), the rise of SDT modeling in the eyewitness identification literature has played a crucial role in highlighting the positive relationship between confidence and accuracy, a relationship that is often neglected or downplayed in the US legal system.

6 Depending on the model considered, at least one the lure distribution variances will be fixed to 1 without loss of generality. Also, we will set $\mu _{L,i} = - \mu _{T,i}$ across all models. According to this parametrization, the origin of the latent strength scale is exactly between the target and lure distributions. Our motivation for choosing this specific parameterization will be made clear later on.

7 Alternatively, one could have specified different response criteria for the different sequences. The problem with that approach is that it would introduce *fifteen* additional parameters instead of just two.

8 The mismatch between the observed and predicted conditional proportions in Fig. 7 can be attributed to the sampling variability in the data. As can be seen in Table 1, the frequency of “yes” responses post-target rejection are rather small (see the frequencies in italic).

9 This constraint did not include the effect of target rejections; i.e., we did not impose any constraints on $\tau ^*_{0,i,h}$.

10 Please note that this is not strictly true in Wilson et al.’s (2019) studies, as their lineup procedure did not enforce a stopping rule. But as mentioned earlier, in the present analysis we are only considering people’s judgments up to their first “yes” response (i.e., a stopping rule was emulated).

11 As discussed in detail by Davis-Stober (2009), the sampling distribution of the $G^2$ statistic, when testing linear order constraints such as monotonicity, follows a mixture of $\chi ^2$ distributions. Instead of determining the weights of this mixture, we simply note that the most conservative mixture distribution, which corresponds to a $\chi _{\text {df}=6}$ distribution, has a critical ($p = .05$) value of 12.59.

12 There is no need to extend the other models, given that confidence ratings have no say on the hypotheses that they were designed to test (e.g., does the response criterion change across sequence positions?). However, there is the possibility that that allowing response criteria to vary freely across sequence positions compromises our ability to reliably detect differences in discriminability along the same variable. We will address (and dismiss) this concern later on.

13 When inspecting model (mis)fits in terms of ROCs, keep in mind that they only capture a small portion of the overall data, which the models attempt to fit in their entirely. For reference, only twelve of the forty-nine cells (24.4%) in each of the datasets reported in Table 1 are included the ROCs.

14 To some degree, these misfits are due to the way that we streamlined the response criteria, using $\delta$, $\lambda$, $\omega$, $\eta$, and $\gamma$. But given that this approach did not yield any systematic misfits (see Kellen & Singmann, 2016), we saw no reason to discard it, especially when an alternative approach would likely explode the number of free parameters.

15 Under the present model parametrization, $d_a = \sqrt{2}\frac{2\mu _T}{\sqrt{1 + \sigma ^2_T}}$ (see Simpson & Fitter, 1973).

16 The equivalence of discriminability across groups was tested by constraining both $\mu _T$ and $\sigma ^2_T$. In turn, the equivalence of response criteria was tested by constraining all six $\tau _0$ as well as $\delta$ and $\lambda$.

17 To understand the loss of information implied, note that the uncensored binary response sequence data from Wilson et al. (2019) can be represented in terms of seven joint multinomial distributions with sixty-four categories each, for a total of 441 degrees of freedom. In contrast, Dunn et al. (2022) represented the data in terms of $7\times 6 = 42$ joint binomial distributions, for a total of just 42 degrees of freedom.

18 This structuring of the data still assumes all cases in which a “yes” response was previously made as i.i.d. This strikes us as a small price to pay given that we distinguish them from cases in which no such response has been made.

19 Please note that this reduction in discriminability after the first “yes” response does not negate the overall improvements coming from removing the stopping rule (see Table 5). Both findings can coexist in perfect harmony. It is easy to see why: Even if discriminability is reduced after the first “yes” response, it is still quite likely that the target, when encountered later on, will be the most familiar out of all the faces in the sequence.
